# Characterization of various cell lines from different ampullary cancer subtypes and cancer associated fibroblast-mediated responses

**DOI:** 10.1186/s12885-016-2193-5

**Published:** 2016-03-08

**Authors:** Zon Weng Lai, Louisa Bolm, Hannah Fuellgraf, Martin L. Biniossek, Frank Makowiec, Ulrich Theodor Hopt, Martin Werner, Tobias Keck, Dirk Bausch, Claudio Sorio, Aldo Scarpa, Oliver Schilling, Peter Bronsert, Ulrich Friedrich Wellner

**Affiliations:** Institute of Molecular Medicine and Cell Research, University of Freiburg, Freiburg, Germany; Clinic for Surgery, UKSH Campus Lübeck, Lübeck, Germany; Department of Pathology, University Medical Center Freiburg, Freiburg, Germany; Clinic for General and Visceral Surgery, University Medical Center Freiburg, Freiburg, Germany; Dipartimento di Patologia, Universita di Verona, Verona, Italy; BIOSS Centre for Biological Signaling Studies, University of Freiburg, Freiburg, Germany; German Cancer Consortium (DKTK), German Cancer Research Center (DKFZ), D-69120 Heidelberg, Germany; Comprehensive Cancer Center Freiburg, Freiburg, Germany; Klinik für Chirurgie, Ratzeburger Allee 160, 23562 Lübeck, Germany

**Keywords:** Ampullary adenocarcinoma, Fibroblast, Differentiation, Intestinal, Pancreatobiliary, Dysplastic, Cell proliferation, Cell invasion

## Abstract

**Background:**

Ampullary cancer is a relatively rare form of cancer and usually treated by pancreatoduodenectomy, followed by adjuvant therapy. The intestinal subtype is associated with markedly improved prognosis after resection. At present, only few cell lines are available for in vitro studies of ampullary cancer and they have not been collectively characterized.

**Methods:**

We characterize five ampullary cancer cell lines by subtype maker expression, epithelial-mesenchymal transition (EMT) features, growth and invasion, drug sensitivity and response to cancer-associated fibroblast conditioned medium (CAF-CM).

**Results:**

On the basis of EMT features, subtype marker expression, growth, invasion and drug sensitivity three types of cell lines could be distinguished: mesenchymal-like, pancreatobiliary-like and intestinal-like. Heterogeneous effects from the cell lines in response to CAF-CM, such as different growth rates, induction of EMT markers as well as suppression of intestinal differentiation markers were observed. In addition, proteomic analysis showed a clear difference in intestinal-like cell line from other cell lines.

**Conclusion:**

Most of the available AMPAC cell lines seem to reflect a poorly differentiated pancreatobiliary or mesenchymal-like phenotype, which is consistent to their origin. We suggest that the most appropriate cell line model for intestinal-like AMPAC is the SNU869, while others seem to reflect aggressive AMPAC subtypes.

**Electronic supplementary material:**

The online version of this article (doi:10.1186/s12885-016-2193-5) contains supplementary material, which is available to authorized users.

## Background

Posing a challenge to clinical and pathological assessment and diagnosis, ampullary adenocarcinoma (AMPAC) involves a wide range of histological differentiation as well as complex anatomic localization [1, 2]. Besides pancreatic, duodenal and distal bile duct adenocarcinomas, the World Health Organization classification system assesses AMPAC as one of the tumors of the periampullary region [3]. In contrast to pancreatic ductal adenocarcinoma (PDAC) clinicopathological studies indicate favorable survival prognosis, lower TNM stage and minor lymph node involvement in AMPAC [1, 4, 5].

Histological differentiation of AMPAC has a strong impact on patient prognosis, as intestinal differentiation is associated with improved survival in comparison to the pancreatobiliary subtype [6, 7]. In pathological routine of hematoxylin-eosin (H&E) staining, difficulties often arise in regards to precise determination of either intestinal or pancreatobiliary type. In fact, histologic subtype is not a black-and-white scale but rather a continuum, in which tumors exist with mixed differentiation. This has led some authors to suggest a “forced binary approach” of subtype assessment [5]. For AMPAC, studies have previously shown that tumor expressing Cytokeratin 20 (KRT 20) correlate to intestinal type, while tumor lacking KRT 20 correlate to pancreatobiliary type [8], Caudal type homeobox 2 (CDX2) positive staining correlate to intestinal type [9, 10], and cytokeratin 7 (KRT 7) is expressed in the majority of pancreatobiliary and a minority of intestinal specimens [11, 12]. Given that there is an existing demand for the development of further prognostic profiles of AMPAC differentiation [13, 14], KRT 20, CXD2 and KRT 7 qualify as potential immunohistochemical markers to determine survival prognosis [6, 15].

The cellular plasticity phenomenon of epithelial-mesenchymal transition (EMT) has become an extensively studied biological concept to explain local tumor invasion and metastasis [16]. Recently, so-called tumor budding has been interpreted as a clinical correlation for partial epithelial-mesenchymal transition at the tumor-stromal interface [17], and has also been previously shown to be a strong prognostic factor in AMPAC [18].

With recent large scale screening methods to demonstrate relevant correlations between the in vitro characteristics in of cell lines and clinical tumor biology [19–22], there has been increasing interests in cell line panels for the development of personalized treatment. However, only few cell lines from human ampullary cancer have been reported thus far, and to our best knowledge, no study has attempted to characterize these collectively [23–26]. Furthermore, the interaction between AMPAC cells of varying differentiation and stromal cells, such as cancer associated fibroblasts (CAF), has yet to be investigated. This study aims to characterize the five AMPAC cell lines with respect to subtype, EMT markers and as well as tumor-stroma interaction.

## Methods

### Ethics, clinicopathologic assessment and immunohistochemistry

Ethics board approval was obtained from the institutional ethics board of the University of Freiburg (ref 13/11). Written informed consent was obtained from patients for the use of tumor tissue for cell culture experiments. For retrospective histopathologic study, patients who underwent surgical treatment for AMPAC were identified from an in-house curated clinical database. A standard pathology protocol was applied for pancreatoduodenectomy specimens and clinicopathologic case review were performed as previously described [6]. Only cases with sufficient formalin-fixed and paraffin-embedded tissue left for re-assessment were included for the study. Immunohistochemical staining for KRT7, KRT20, CDX2, ZEB1 and E-Cadherin was performed as previously described [6, 27]. Tumor budding was quantified at the invasive front according to a protocol previously established from pancreatic cancer [27]. CAF activation grade was classified according to Ha et al [28] as high (immature stroma) or low (mature stroma). Mature tumor stroma was defined as tumor stroma including fibroblasts with small spindle cell morphology, a thin and wavy body-structure and a symmetric/parallel orientation. Immature tumor stroma included fibroblasts with plump spindle-shaped cell morphology, a prominent nucleus with prominent nucleoli and with randomly a spatial orientation. Tumor stroma was evaluated in two fields of twenty fold magnification. A value of more than 50 % immature fibroblasts of all fibroblasts were was considered as immature tumor stroma phenotype.

### Clinical statistics and hierarchical clustering

Data collection and statistical analyses were performed operating with IBM SPSS Version 21 (SPSS Inc, Chicago, IL) and MedCalc Version 14 software (Medcalc bvba, Ostend, Belgium). Precise scaling for the different variables were expressed as median (and range), categorical parameters (cross-tabulation and percentages), and survival data (Kaplan-Meier method). For statistical testing, Spearman rank correlation and log rank test were employed. For hierarchical clustering and generation of heat-map images, Multi Experiment Viewer (MEV, www.tm4.org) [29] was used. To generate MEV dataset, gene or antigen expression levels were expressed as relative values with the maximum level defined as 100 %, and samples were named according to tumor subtype (INT intestinal, PB pancreatobiliary, POOR poorly differentiated) or cell line. Upon loading to MEV, gene/row normalization and hierarchical clustering (HCL) was performed [30] and heat-map images of the HCL tree diagrams generated for visual interpretation.

### Cell culture and treatment with CAF-conditioned medium

Ampullary carcinoma cell lines (MDA-AMP7, AVC1, RCB1280, SNU478, and SNU869) were obtained from Prof. Frazier (MDA-AMP7, MD Anderson Cancer Center, Houston/Texas, USA) [23], Prof. Sorio (AVC1, Institute of Pathology, University of Verona, Italy) [26], the RIKEN Cell Bank (RCB1280, http://cell.brc.riken.jp/en/rcb, Japan) and the Korean Cell Line Bank (SNU478 and SNU869, http://cellbank.snu.ac.kr/english/index.php, Seoul, South Korea) [31]. PANC1 cells were purchased from the American Type Culture Collection (ATCC, www.atcc.org). All cells were cultured at 37 °C in 5 % CO2 atmosphere in DMEM high glucose medium with GlutamaX (Life Technologies #10566-032) containing 10 % FBS (Life Technologies Standard FBS #10500-064).

Cancer associated fibroblasts (CAF) were isolated by Bachem’s outgrowth method [32] from a human ampullary adenocarcinoma (pancreatobiliary subtype) resected by pancreatoduodenectomy, and cell type and purity assessed by morphology and immunofluescent staining for Vimentin and Pan-Cytokeratin as described [17]. CAFs were expanded in 75 cm^2^ cell culture flasks to 70 % confluence and cryopreserved at -80 °C in standard freezing medium containing DMSO. Only CAF up to passage six were used.

For generation of CAF conditioned medium (CAF-CM), CAFs were grown until 70 % confluence, before switching to fresh medium (DMEM 10 % FCS) and incubated for 72 h. CAF-CM was then removed from the CAFs, centrifuged at 1000 rpm for 5 min, sterile filtered (0.22 μm), and transferred to AMPAC cells, which were previously seeded in standard culture flasks for one day prior and washed with PBS. Fresh CAF-CM was added every three days. AMPAC cells were cultured in CAF-conditioned medium for 5–7 days until 70 % confluence and CAF-CM treated and control cells were harvested by cell scraper after three times rinsing with PBS, centrifuged and pelleted at 1000 rpm for 5 min.

### Gene expression analysis, cell invasion and growth assay

Immunofluorescence of cultured cells and real time PCR for measurement of mRNA expression was performed as previously described [17]. Primer sequences were Actin beta (ACTB, GCCCTGAGGCACTCTTCCA, TTGCGGATGTCCACGTCA) E-Cadherin (CDH1, GTCCTGGGCAGAGTGAATTT, GACCAAGAAATGGATCTGTGG), Zinc finger E-box binding homeobox-1 (ZEB1, AAGAATTCACAGTGGAGAGAAGCCA, CGTTTCTTGCAGTTTGGGCATT), Caudal type homeobox 2 (CDX2, CTGGAGCTGGAGAAGGAGTTTC, ATTTTAACCTGCCTCTCAGAGAGC), Cytokeratin 7 (KRT7, TGCTGAAGAAGGATGTGGATGCTGC, TCTGGGACTGCAGCTCTGTCAACT) and Cytokeratin 20 (KRT20, GCGACTACAGTGCATATTACAGACAA, GCAGGACACACCGAGCATTT). Cultrex BME Cell Invasion Assay (Trevigen #3465-096-K) was performed according to the manufacturer’s instructions as described [33]. For assessment of CAF effect on AMPAC cell invasiveness, CAF were seeded in the lower chamber in standard culture medium and left until 70 % confluent after 3–6 days, while control wells contained no CAFs. AMPAC cells were then seeded into the upper chamber wells in DMEM medium with 0.1 % FBS and matrigel transmigration measured after three days. Cell growth was measured using 3-(4,5-dimethylthiazol-2-yl)-2,5-diphenyltetrazolium bromide (MTT) assay as described [33]. For growth inhibition, low-dose Gemcitabine 40nM (Eli Lilly Co, Indianapolis, USA) was added to the culture medium one day after seeding 1000 cells per well into 96 well standard culture plates, and cell growth quantified after three days of treatment. Herein the pancreatic cancer cell line PANC1 served as a Gemcitabine-resistant model [34]. Similarly, all cells were incubated in Gemcitabine (100 nM, 1 μM or 10 μM) one day after seeding 20 000 cells per well into 24 well standard culture plates, and cell growth quantified after three days of treatment.

### Quantitative proteome comparison

Cell pellets from harvesting were lysed using buffer containing 20 mM Tris, pH 7.5, 150 mM NaCl, 1 % (v/v) Triton X-100 in the presence of protease inhibitors protease inhibitors (5 mM ethylene diamine tetraacetic acid, 10 μM (2S, 3S)-trans-epoxysuccinyl-L-leucylamido-3-methylbutane ethyl ester, 1 mM phenylmethanesulfonyl fluoride). Lysates were kept on ice for 30 min with intermittent inverting to ensure proper lysis. Lysates were centrifuged for 10 min at 16 000 × g and 4 °C. Protein concentration of lysates was determined using bicinchoninic acid assay. Proteins were precipitated using ice-cold acetone and trypsinized (Worthington Biochemical Corp., Lakewood, NJ, USA). Cysteine residues were reduced and alkylated, followed by dimethylation of peptide N-termini and lysine residues. Samples from control treated cells were isotopically labeled using 40 mM ^12^COH_2_ formaldehyde (Sigma-Aldrich, Steinheim, Germany) while samples from fibroblast conditioned medium were isotopically labeled using 40 mM ^13^COD_2_ formaldehyle (Cambridge Isotope Laboratories, Andover, MA, USA) [35, 36], both in the presence of 40 mM sodium cyanoborohydride for 16 h at room temperature. Excess formaldehyde was quenched using 20 mM glycine. Samples were combined in a 1:1 (w/w) ratio manner and desalted using a reversed phase C18 Sep-Pak SPE column (Waters, Milford, MA, USA). Samples were subsequently fractionated using high performance liquid chromatography, coupled to a strong cation exchange column (PolyLC, Columbia, MD, USA). Buffer A consisted of 5 mM KH_2_PO_4_ and 25 % (v/v) acetonitrile (pH 2.7), and buffer B consisted of 5 mM KH_2_PO, 1 M KCl, and 25 % acetonitrile (pH 2.7). Peptides were eluted in a linear gradient with increasing concentration of buffer B. Resulting fractions were collected, desalted using self-packed C18 STAGE tips (Empore, St. Paul, MN, USA) [37], and analyzed by mass spectrometry.

### Mass spectrometry and data processing

Samples were analyzed on an Orbitrap XL (Thermo Scientific, Bremen, Germany) or an Orbitrap Q-Exactive plus (Thermo Scientific) mass spectrometer. The Orbitrap XL was coupled to an Ultimate3000 micro pump (Thermo Scientific). Buffer A was 0.5 % (v/v) acetic acid, buffer B 0.5 % (v/v) acetic acid in 80 % acetonitrile (HPLC grade). Liquid phases were applied at a flow rate of 300 nl/min with an increasing gradient of organic solvent for peptide separation. Reprosil-Pur 120 ODS-3 (Dr. Maisch) was used to pack column tips of 75 μm inner diameter and 11 cm length. The MS was operated in data dependent mode and each MS scan was followed by a maximum of five MS/MS scans. The Q-Exactive plus mass spectrometer was coupled to an Easy nanoLC 1000 (Thermo Scientific) with a flow rate of 300 nl/min. Buffer A was 0.5 % formic acid, and buffer B was 0.5 % (v/v) formic acid in acetonitrile (water and acetonitrile were at least HPLC gradient grade quality). A gradient of increasing organic proportion was used for peptide separation (5–40 % (v/v) acetonitrile in 80 min). The analytical column was an Acclaim PepMap column (Thermo Scientific), 2 μm particle size, 100 Å pore size, length 150 mm, inner diameter 50 μm. The mass spectrometer operated in data dependent acquisition mode with a top ten method at a mass range of 300–2000 Da.

LC-MS/MS data were analyzed using X! Tandem (Version 2013.09.01) [38] in conjunction with PeptideProphet [39] using a 5 % peptide false discovery rate and a decoy search strategy, and ProteinProphet [40] at a protein false discovery rate of 1 %. The protein database was composed of annotated human UniProt protein sequences (without isoforms, downloaded on on November 26, 2013 with 20,240 real protein entries), combined with a randomized and a reversed decoy database X! Tandem parameters included: precursor mass error of ± 10 ppm, fragment ion mass tolerance of 20 ppm (Q-Exactive) or 0.4 Da (Orbitrap XL), tryptic specificity with no missed cleavage, static residue modifications: cysteine carbamidomethylation (+57.02 Da), as well as lysine and N-terminal dimethylation (^12^COH_2_ formaldehyde, +28.03 Da; ^13^COD_2_ formaldehyde, +34.06 Da). For relative peptide and protein quantification, XPRESS [41] was used. Mass tolerance for quantification was ± 0.015 Da (for Orbitrap XL), or ± 20 ppm (for Q-Exactive Plus). Ratios of each dataset were calculated as treated cells over control. The mass spectrometry proteomics data have been deposited to the ProteomeXchange Consortium [42] via the PRIDE partner repository with the dataset identifier PXD002657 (username: reviewer66711@ebi.ac.uk password: TdyZ2Yle). XPRESS data was log_2_ transformed, resulting in fold-change values. Proteins were considered as being differentially regulated if the proteins are consistently expressed minimum three out of five cell lines with a fold-change value of greater than 0.58 (differential abundance equivalent to 50 %). The list of affected proteins was submitted to STRING database version 9.1 [43]. Predicted functional connections among proteins are based on the following criteria: neighborhood, co-expression, gene fusion, experiments, co-occurrence, databases and text-mining. QIAGEN’s Ingenuity® Pathway Analysis (IPA®, QIAGEN Redwood City, www.qiagen.com/ingenuity) was further used to derive and visualize biological themes that were significantly associated to affected proteins identified from mass spectrometry analyses.

## Results

### Clinical survival analysis and biologic correlation

Clinicopathologic review resulted in the inclusion of *n* = 39 patients (17 women, 22 men) treated by pancreatoduodenectomy for AMPAC from 2001 to 2011. Median tumor size was 20 mm, about half of tumors were of T1/T2 stage and 59 % had locoregional lymph node metastasis, with a lymph node ratio of 0.10 or more in 44 %. Most tumors displayed low grading (78 % G1 or G2), microscopic lymphangiosis was common (49 %), perineural invasion found in only 33 %, and hemangiosis rare (5 %). There were only three cases with positive surgical resection margin (Table 1).

**Table 1:** Baseline parameters

Parameter	Condition	n or median	% or range	*p*-value
n		39	100 %	-
Age (years)		64	36–84	0.063^a^
Sex	Female	17	44 %	0.918
	Male	22	56 %	
Death during follow-up		13	33 %	-
Follow-up (months)		27	1–116	-
Tumor size (mm)		20	2–320	0.497
T stage	T1/2	19	49 %	0.140
	T3/4	20	51 %	
Lymph node metastasis		23	59 %	0.112
Lymph node ratio > = 0.10		17	44 %	0.003
Lymphangiosis		19	49 %	0.006
Hemangiosis		2	5 %	0.778
Perineural invasion		13	33 %	0.407
Tumor grade	G1-2	28	72 %	0.439
	G3-4	11	28 %	
Positive margin		3	8 %	0.000
Subtype	INT	18	46 %	0.030^b^
	PB	17	44 %	
	POOR	4	10 %	

*p* values derived from two-sided Logrank test. ^a^cutoff at median, ^b^test for INT vs non-INT subtype

*Abbreviations*: *INT* intestinal subtype, *PB* pancreatobiliary subtype, *POOR* poorly differentiated adenocarcinoma

Using a forced-binary approach [5], histopathological subtypes found were intestinal (46 %), pancreatobiliary (44 %) and poorly differentiated (10 %). At a median follow-up of 27 months, only 13 patients had died and median survival was not reached, giving a mean survival estimate of 74 months. Patients with positive surgical margin status suffered poor prognosis (median survival eight months, *p* = 0.000). Biological factors that showed significant prognostic impact (*p* < 0.05) include intestinal subtype, lymph node radio and lymphangiosis. Correlation analysis between these prognostic factors disclosed a negative correlation between intestinal subtype and lymph node ratio, and a positive correlation of lymphangiosis with lymph node ratio (Table 2).

**Table 2:** Correlation matrix for prognostic factors in ampullary adenocarcinoma

		INT	PB	LNR	L	R
INT	CC		-0.81	-0.59	-0.18	-0.27
	p		.000	.000	.267	.100
PB	CC	-0.81		0.48	0.07	0.33
	p	.000		.002	.653	.041
LNR	CC	-0.59	0.48		0.52	0.21
	p	.000	.002		.001	.194
L	CC	-0.18	0.07	0.52		0.30
	p	.267	.653	.001		.067
R	CC	-0.27	0.33	0.21	0.30	
	p	.100	.041	.194	.067	

CC and *p* values derived from two-sided Spearman rank correlation

*Abbreviations*: *CC* correlation coefficient, *INT* intestinal subtype, *PB* pancreatobiliary subtype, *LNR* lymph node ratio, *L* lymphangiosis, *R* margin positive resection

**Fig. 1:**
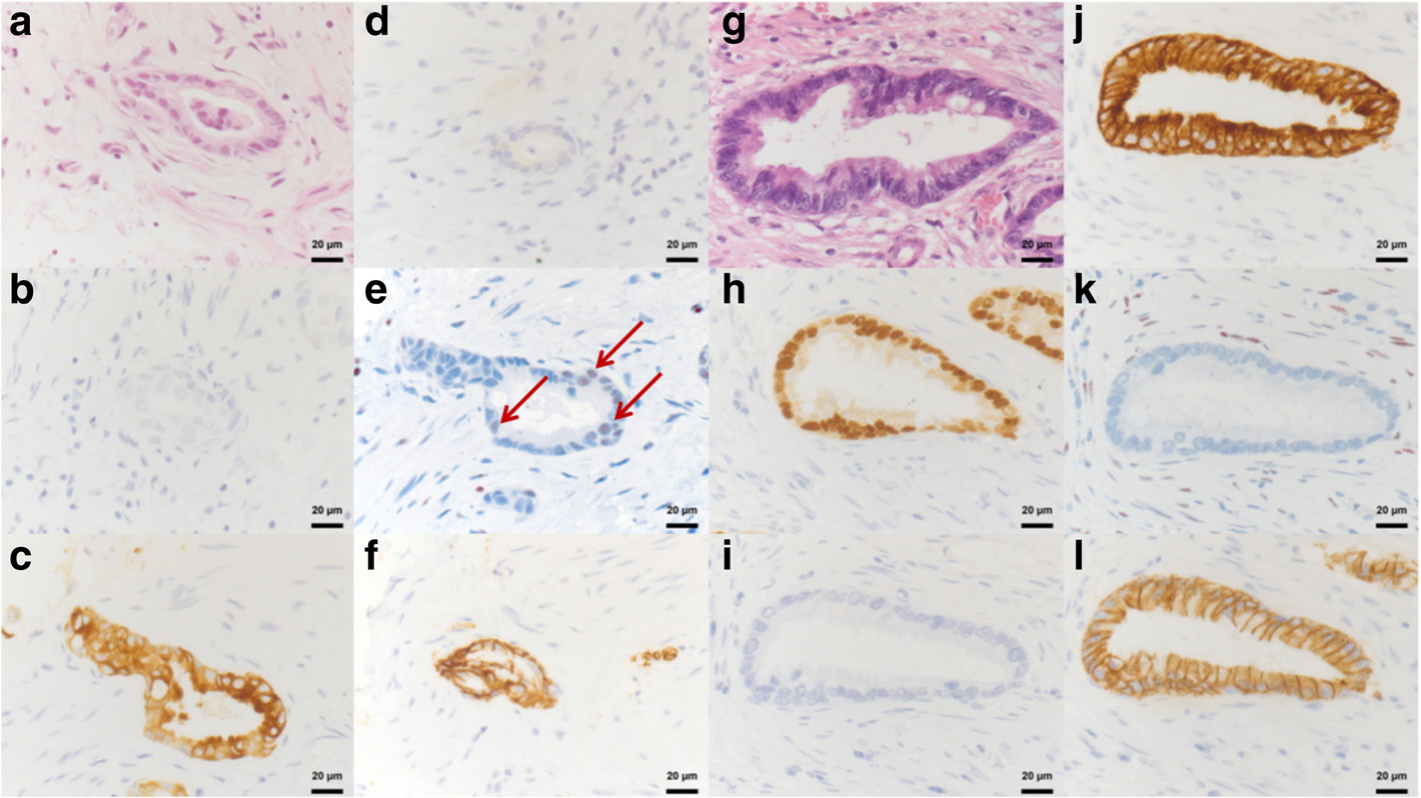
Immunohistochemical staining of ampullary cancer. Subtype (**a**-**d**; **g**-**j**) and EMT (**e**&**f**; **k**&**l**) histomorphologoical and immunohistological analysis for the intestinal (**a**-**f**) and pancreatobiliary (**g**-**l**) AMPAC subtype, taken at 40-fold magnification. HE (**a**&**g**) staining representing the pancreaticobilliary (**a**) subtype with cuboidal formed columnar tumor cells and rounded nuclei, membranous KRT7 (**c**) positivity, CDX2 (**b**) and KRT20 (**d**) negativity and the intestinal type (**g**) with pseudostratified mucin producing glandular epithelium, elongated hyperchromatic and pseudostratified nuclei, nuclear CDX2 (**h**) and membranous KRT20 (**j**) positivity and KRT7 negativity (**i**). Nuclear ZEB1 expression (**e**, *red arrows*) is linked with a immunohistological membranous to cytoplasmatic Ecad shuttling. Contrary absent ZEB1 expression is immunohistological accompanied with membranous Ecad expression. Abbreviation: *EMT* Epithelial-Mesenchymal-Transition; *AMPAC* Ampullary Adenocarcinoma; *HE* Hematoxylin-Eosin, *KRT* Cytokeratin, *CDX2* Caudal Type Homeobox 2, *ZEB1* Zinc finger E-box binding homeobox 1, *Ecad* E-Cadherin

**Fig. 2:**
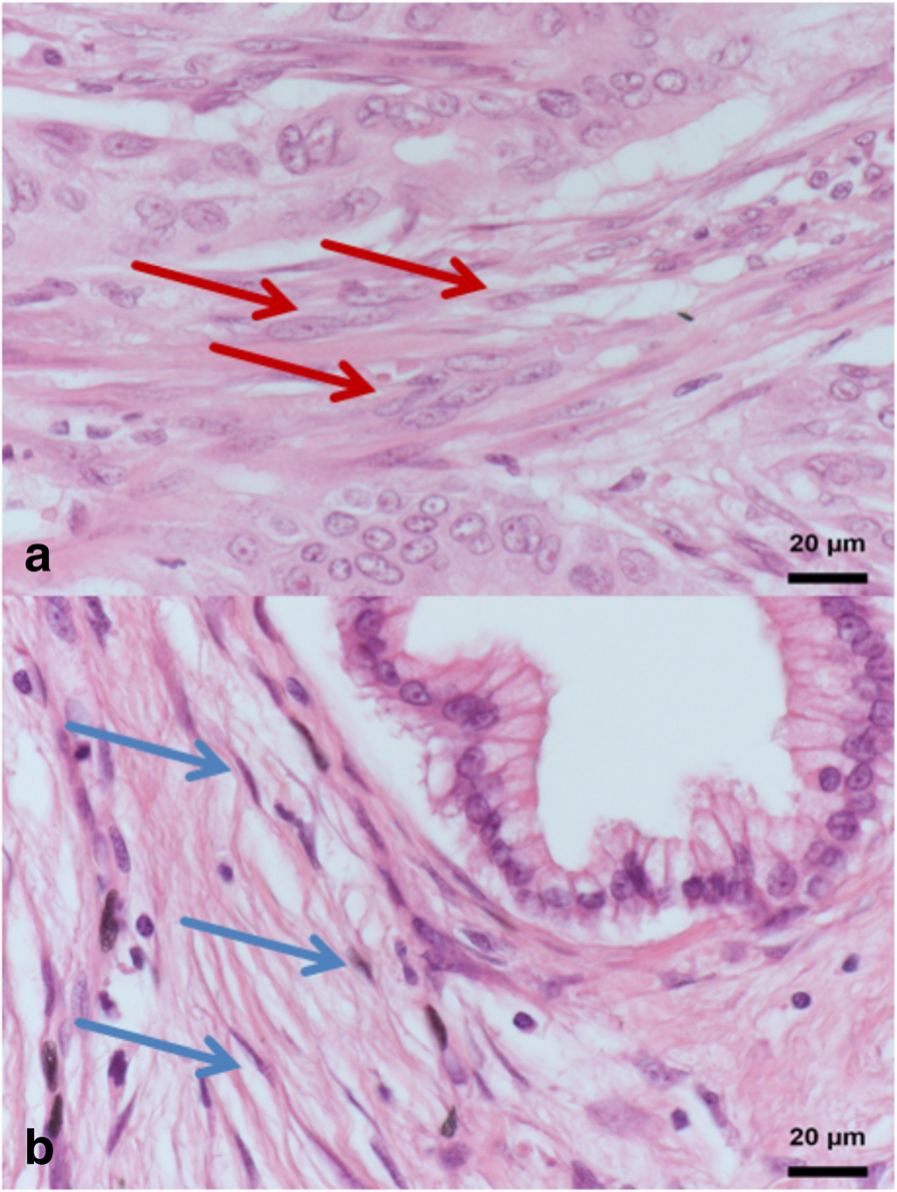
CAF activation grade in AMPAC, represented by HE staining taken at 40-fold magnification. **a** Immature tumor stroma with plump spindle-shaped cell morphology, prominent nucleus, prominent nucleoli (red arrow) and with randomly a spatial orientation. **b** Mature tumor stroma with small spindle cell morphology, a thin and wavy body-structure (blue arrow) and a symmetric/parallel orientation. Abbreviation: AMPAC = Ampullary Adenocarcinoma; HE = Hematoxylin-Eosin

To further assess the biology of the intestinal differentiation, immunohistochemical staining was performed for KRT 7, KRT 20 and CDX2 for confirmation of phenotype, as well as E-Cadherin and ZEB1 for assessment of epithelial-mesenchymal transition (EMT) (Fig. 1). Tumor stroma was assessed by morphologic CAF activity grading. In agreement with previous reports, our results show high KRT7 and E-Cadherin expressions, low CDX2 and KRT20 expressions, and occasional ZEB1 and Vimentin expressions in the non-intestinal subtypes (pancreatobiliary and poorly differentiated). In contrast to Vimentin, the variation in ZEB1 expression level were rather high. In addition, tumor budding and CAF grade are elevated in pancreatobiliary type cancers. Furthermore, intestinal type tumors showed significantly reduced ZEB1 expression in tumor cells, higher tumor budding at the invasive front as well as reduced CAF activation grade compared to non-intestinal tumors (Table 3) (Fig. 2a and b). This has led us to investigate the interaction between CAF and tumor cells in vitro to evaluate causality of these associations.

**Table 3:** Tumor biologic factors correlating with the intestinal subtype

Parameter	Subtype			Correlation with INT	
	INT	PB	POOR	CC	*p*
CK7 %	70 (0–100)	90 (5–100)	40 (0–90)	-0.24	0.148
CK20 %	90 (0–100)	5 (0–100)	2.5 (0–10)	0.47	0.003
CDX2 %	90 (20–100)	10 (0–80)	45 (0–100)	0.69	0.000
ZEB1 %	0 (0–2)	0 (0–16)	2 (0–3)	-0.32	0.046
VIM %	0 (0–10)	0 (0–10)	0 (0–0)	0.02	0.914
ECad %	100 (50–100)	100 (75–100)	75 (50–100)	0.03	0.869
Budding	8 (0–35)	15 (2–46)	43 (6–66)	-0.46	0.003
CAF	1 (0–2)	2 (0–2)	0.5 (0–1)	-0.34	0.035

CC and *p* values derived from two-sided Spearman rank correlation. % expression in percentage of tumor cells, Tumor budding measured as number of tumor buds per HPF (high power field)

*Abbreviations*: *INT* intestinal, *PB* pancreatobiliary, *POOR* poorly differentiated, *CC* correlation coefficient, *KRT* cytokeratin staining, *CDX2* caudal type homeobox 2, *ZEB1* zinc finger and homeobox 1, *ECad* E-Cadherin, CAF cancer associated fibroblast activation

### Culture and characterization of ampullary cancer cell lines and CAF

**Fig. 3:**
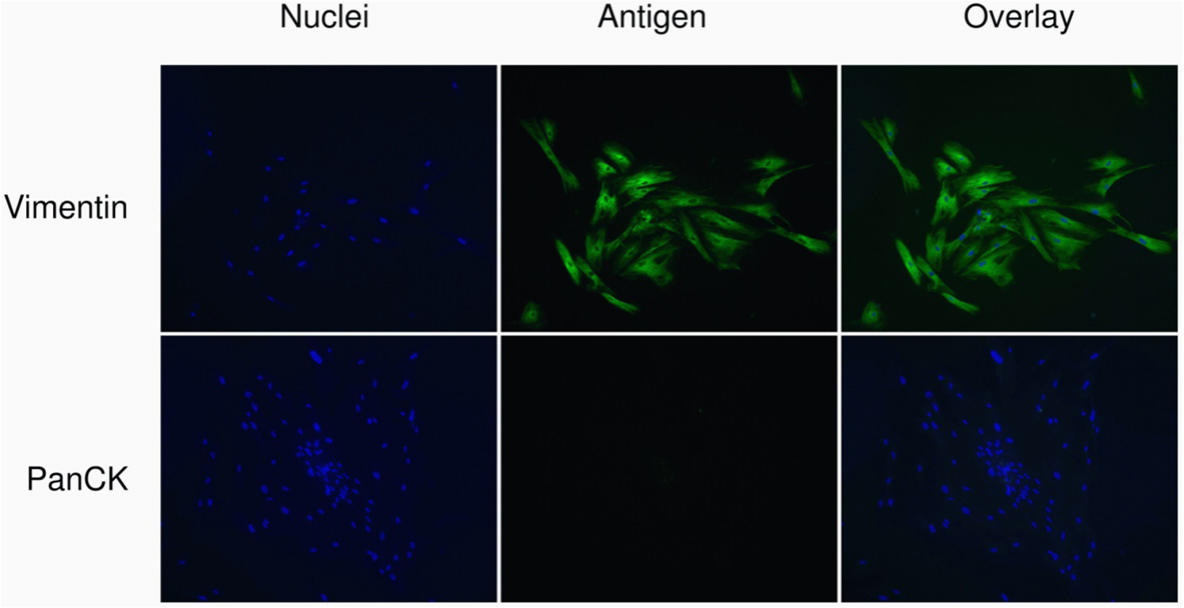
Cancer associated fibroblasts isolated from ampullary cancer. Cancer associated fibroblasts (CAF) were isolated from human ampullary cancer by the outgrowth method. Immunofluorescent staining confirms strong expression of Vimentin (VIM) and typical spindle-like morphology, as well as lack of Pan-Cytokeratin (PanCK) staining

CAFs were isolated from a human ampullary cancer explant using Bachem’s outgrowth method [32]. Cell type and purity was confirmed by typical morphology using phase-contrast microscopy, in which strong vimentin expression and lack of Pan-Cytokeratin staining in immunofluorescence were observed (Fig. 3). Literature review revealed eight reported cell lines derived from AMPAC (Table 4): two cell lines were derived from distant metastases (MDA-AMP7 and RCB1280), one cell line from a poorly differentiated primary tumor with signet ring cell features (SNU478), and one from a moderately differentiated AMPAC arising in a villous adeoma (AVC1). Therefore most cell lines stem from tumors with aggressive biological features, leaving only one cell line derived from a well-differentiated AMPAC (SNU869). At present, no information on AMPAC subtype or patient follow-up has been reported. One cell line already ceased (UKEAC-99, personal communication with author), and the rest was obtained from the cell line banks or authors (see methods). Five of seven cell lines were successfully cultured in standard culture medium (DMEM 10 % FCS) and are used for further experiments. Three cell lines (RCB1280, RCB1281, RCB1282) are derived from the same patient, indicating successful culture of one cell line from each reported patient.

**Table 4:** Ampullary cancer cell lines reported in the literature

Cell line	Primary tumor	Cell line derived from	Histopathology/differentiation	TNM stage	Kras status	Further characterization	Patient follow-up	Reference
*Cell lines successfully grown in standard culture medium*								
MDA-AMP7	AMPAC	Peritoneal metastasis	Not reported	rpM1	Not reported	Aneuploidy and structural chromosomal mutations	Peritoneal metastases occurred 9 months after resection of primary tumor	Frazier et al. (Frazier et al. 1992)
AVC1	AMPAC	Primary tumor	Moderately differentiated, arising from villous ampullary adenoma	pT1N0 cM0	Mutated	Tumorigenic in mice, moderate Gemcitabine sensitivity	Perioperative death	Sorio et al. (Sorio et al. 2004)
RCB1280	AMPAC	Liver metastasis	not reported	not reported	Not reported	structural chromosomal mutations	Not reported	RCB
SNU478	AMPAC	Primary tumor	poorly differentiated with signet ring cell features	pN0 (0/5)	Wild type	E-Cadherin wild type but methylated	Not reported	Ku et al. (Ku et al. 2002)
SNU869	AMPAC	Primary tumor	well differentiated with focal papillary differentiation	pN1 (5/10)	Wild type	E-Cadherin wild type and expressed	Not reported	Ku et al. (Ku et al. 2002)
*Other cell lines reported*								
UKEAC-99	AMPAC	Primary tumor	cell line discarded (personal communication with authors)					Peiper et al. (Peiper et al. 2003)
RCB1281	AMPAC	Peritoneal metastasis	cell line derived from same patient as RCB1280					RCB
RCB1282	AMPAC	Lymph node metastasus						RCB

*Abbreviations*: *AMPAC* ampullary adenocarcinoma, *RCB* RIKEN Bioresource Center Cell Bank http://cell.brc.riken.jp/en/

**Fig. 4:**
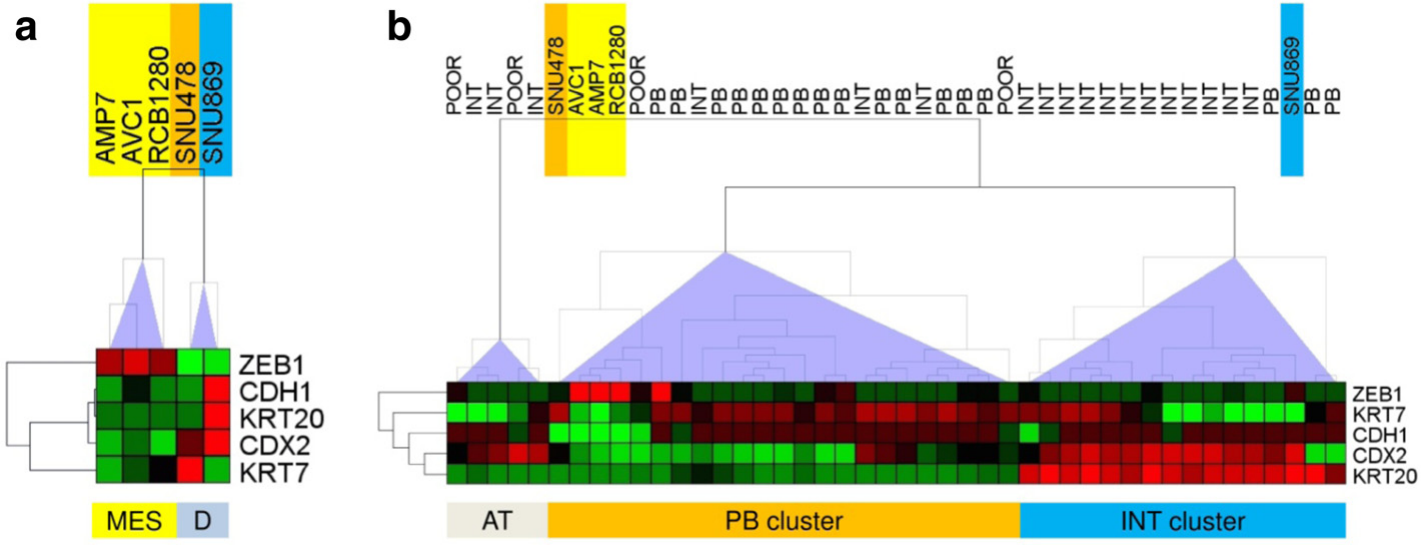
Heatmaps and hierarchical clustering trees of ampullary cancer cell lines and tumors. Relative expression values of EMT (ZEB1, CDH1) and subtype marker genes (KRT7, KRT20, CDX2) were calculated by linear scaling, with maximum expression in cell lines/tumors defined as 100 % and subjected to hierarchical clustering (HCL). **a** HCL tree of the cell lines discloses an mesenchymal-like (MES) and a differentiated (D) cluster of cell lines. MES cells show weak or no expression of subtype marker genes but strong ZEB1 and low E-Cadherin (CDH1) expression. One differentiated cell line displays pancreatobiliary-like (SNU478, orange), the other an intestinal-like marker gene expression pattern (SNU869, blue). **b** HCL tree of tumors and cell lines results in separation of an intestinal (INT), pancreatobiliary (PB) and atypical (AT) cluster. The cell lines are found separated to the PB and INT clusters. For further details see text. Abbreviations: EMT epithelial-mesenchymal transition, ZEB1 zinc finger E-box binding homeobox 1, CDH1 Cadherin 1 = E-Cadherin, KRT Cytokeratin, CDX2 Caudal type homeobox 2, POOR poorly differentiated

The ampullary adenocarcinoma cell lines MDA-AMP7, AVC1, RCB1280, SNU478 and SNU869 were grown in standard culture medium and expression of KRT7, KRT20, CDX2, E-Cadherin and ZEB1 mRNA was measured by real-time PCR. To investigate the heterogeneous expression pattern, we employed unsupervised hierarchical clustering for analysis. Results evidently indicate that three types of expression patterns (Fig. 4a): Cell lines MDA-AMP7, AVC1, RCB1280 displayed an undifferentiated mesenchymal-like expression pattern, characterized by strong expression of the EMT-inducer ZEB1 and low or lacking expression of E-Cadherin and subtype markers KRT7, KRT20 and CDX2. The SNU478 cells exhibited a pancreatobiliary-like expression pattern with strong KRT7 and low KRT20 and CDX2 expression, while the SNU869 cell line showed an intestinal-like expression pattern with strong KRT20 and CDX2, but no KRT7 expression. The latter two cell lines also displayed no or weak expression of the EMT-inducer ZEB1. Interestingly, intestinal-like SNU869 cells is the only cell line to express high amounts of E-Cadherin.

To compare the gene expression pattern of cell lines to the expression pattern in the human AMPAC tumor samples, maximum gene expression was defined as 100 % and relative expression values calculated on a linear scale for each gene. Upon analysis of the relative expression datasets of tumors and cell lines, unsupervised hierarchical clustering revealed two main clusters; intestinal type tumors and pancreatobiliary, respectively (Fig. 4b). Poorly differentiated tumors did not form a distinct cluster, but were found as a sub-cluster of the pancreatobiliary type tumor. Additionally, there was a minor cluster of apparently atypical expression pattern tumors, consisting of three morphologically intestinal-type and two poorly differentiated tumors and characterized by strong CDX2 expression but no KRT20 or KRT2 expression. In regards to the cell lines, the mesenchymal-like cell lines MDA-AMP7, AVC1 and RCB1280 clustered together with a poorly differentiated tumor in the pancreatobiliary cluster, while being closely related to the pancreatobiliary-like SNU478 cell line together with a pancreatobiliary tumor. The intestinal-like cell line SNU869 however was found within the cluster for intestinal tumor, a clear separation from the other cell lines.

### In vitro growth, invasion and chemosensitivity

**Fig. 5:**
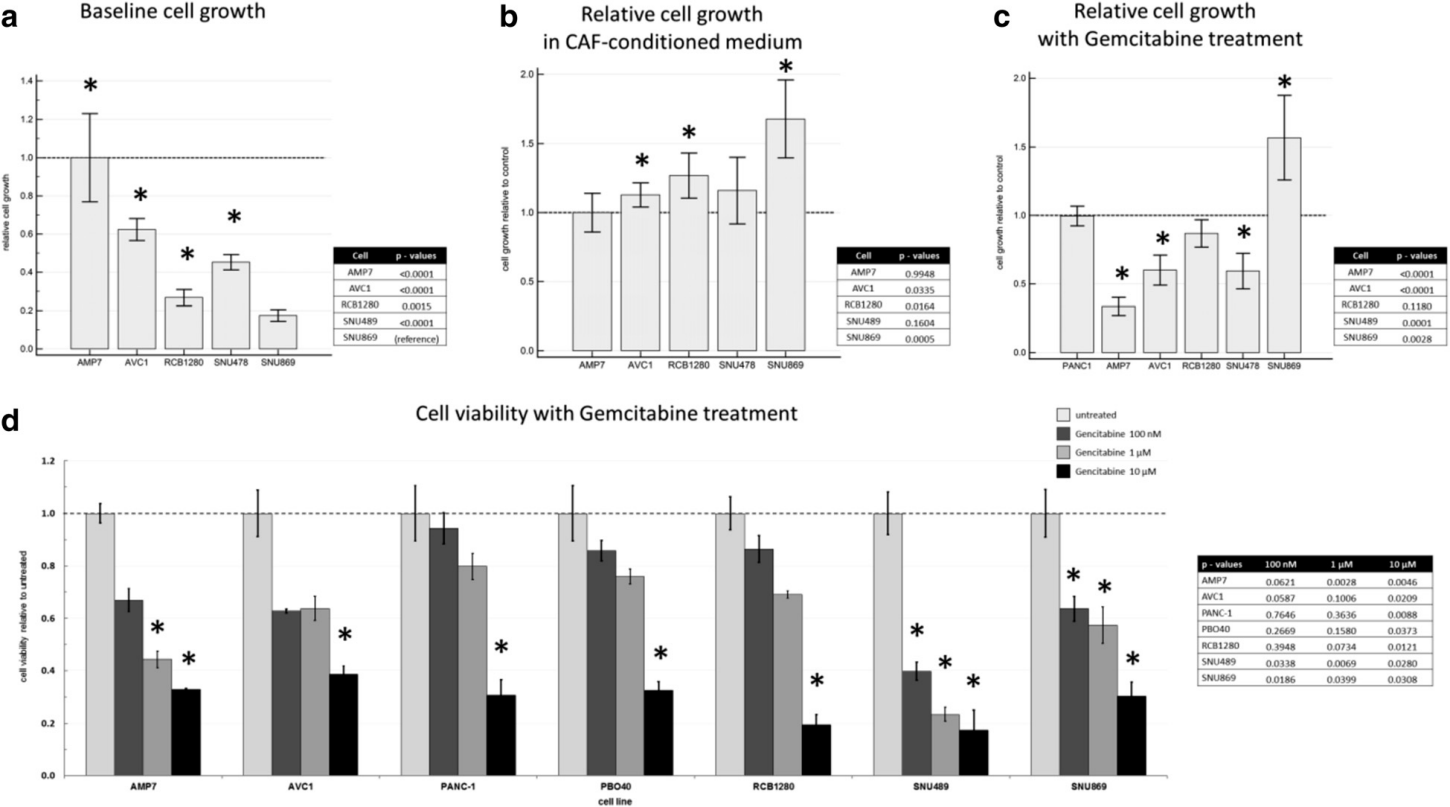
Gemcitabine treatment of ampullary cancer cell lines. Cell viability was quantified by MTT assay. **a** baseline cell growth relative to the fastest growing (MDA-AMP7) cells (**b**) cell growth in CAF-CM relative to controls (**c**) cell growth under low-dose Gemcitabine (40 nM) treatment relative to PANC-1 (**d**) cell viability under varying concentration of Gemcitabine (100 nM, 1 μM or 10 μM) relative to untreated controls. Bar charts depict mean and error bars standard deviation from *n* = 6 measurements, * *p* < 0.05 in two-sided *t*-test compared to untreated control. Abbreviations: MTT 3-(4,5-dimethylthiazol-2-yl)-2,5-diphenyltetrazolium bromide, CAF-CM cancer associated fibroblast conditioned medium

**Fig. 6:**
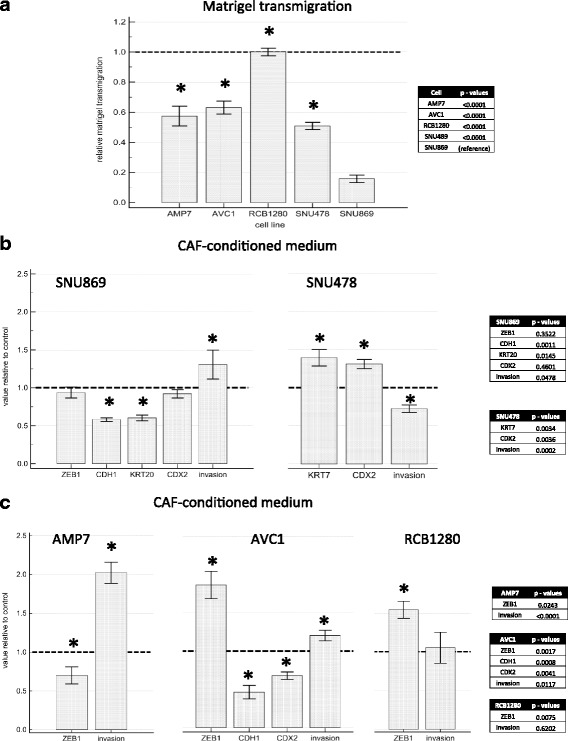
Cell invasion and gene expression changes in ampullary cancer cell lines treated with CAF conditioned medium. Cell invasion was quantified by matrigel transmigration assay and mRNA expression by real time PCR. **a** baseline matrigel transmigration relative to most invasive cell line RCB1280. **b** effect of CAF-CM on intestinal-like cell line SNU869 and pancreatobiliary-like cell line SNU478. **c** effect of CAF-CM on mesenchymal-like cell lines MDA-AMP7, AVC1 and RCB1280. Diagrams depict mean and error bars standard deviation from *n* = 4 measurements, * *p* < 0.05 in two-sided *t*-test compared to untreated control. Abbreviations: AMP7 MDA-AMP7, CAF-CM cancer associated fibroblast conditioned medium, ZEB1 zinc finger E-box binding homeobox 1, CDH1 Cadherin 1 = E-Cadherin, KRT Cytokeratin, CDX2 Caudal type homeobox 2

Following above-mentioned observations, we hypothesized that cell invasion in vitro would reflect the clinical tumor aggressiveness. Baseline cell growth assessed by MTT assay demonstrated that SNU869 cells were the slowest growing among all AMPAC cell lines (Fig. 5a). Also the matrigel transmigration assay confirmed that the mesenchymal-like cell lines had the strongest invasion, followed by the pancreatobiliary-like SNU 478, while the intestinal-like SNU869 cells were far less invasive (Fig. 6a). Low-dose Gemcitabine, a commonly used drug for adjuvant AMPAC treatment [44], significantly inhibited the growth of the mesenchymal- and pancreatobiliary-like AMPAC cells, but not of the intestinal-like cell line, where cell growth was even slightly increased after three days. As a control, growth of PANC1, a pancreatic cancer cell line known to be Gemcitabine resistant [34], was not changed (Fig. 5b). Cell viability of all cell lines in response to higher concentrations of Gemcitabine is also highlighted (Fig. 5d).

### Effects of CAF-conditioned medium on ampullary cancer cells

Given the clinical association of high CAF activation grade in the more aggressive tumors with pancreatobiliary differentiation and EMT features, we further hypothesized that CAF could induce EMT and invasion, as well as subtype shift in cancer cells. To examine the paracrine effect of CAF, ampullary cancer cells were treated with conditioned medium from CAF (CAF-CM) and subjected to growth assay as well as matrigel transmigration assay with CAFs serving as attractant (Fig. 6b and c).

In the intestinal-like cell line SNU869, ZEB1 levels remained unchanged but E-Cadherin expression levels were reduced by approximately 50 %, while cell growth and invasion increased by about 50 % with CAF-CM treatment (Figs. 5b and 6b). The pancreatobiliary-like cell line SNU478 did not express significant changes in level of ZEB1 or E-Cadherin. However, a decrease of matrigel transmigration and no change in growth (Figs. 5b and 6b) were observed in SNU478 cells. Regarding the expression of the subtype marker genes, a marked decrease of KRT20 expression and small but significant decrease in CDX2 expression was observed in the intestinal-like SNU869 cells, while KRT7 and CDX2 expression increased in the pancreatobiliary-like SNU478 cells (Fig. 6b).

Cell growth was slightly enhanced by CAF-CM in the mesenchymal-like cell lines except for the already fast-growing MDA-AMP7 (Fig. 5a and b). Among the mesenchymal-like cell lines, AVC1 was the only cell line expressing measurable amounts of E-Cadherin. Consistent with EMT process, both ZEB1 expression and cell invasiveness were increased while E-Cadherin expression decreased with CAF-CM treatment (Fig. 6c). E-Cadherin was not expressed in the other two mesenchymal-like cell lines, and the high basal ZEB1 expression levels increased in RCB1280 cells but decreased in MDA-AMP7 cells. Nevertheless, matrigel transmigration were increased in MDA-AMP7 cells. RCB1280, as the most invasive of the five cell lines, did not further increase its matrigel transmigration with CAF-CM treatment (Fig. 6c). Regarding subtype markers among the mesenchymal-like cells, only CDX2 was expressed to a relevant but low degree in MDA-AMP7 cells, and further decreased with CAF-CM treatment (Fig. 6c).

### Proteomic analysis of CAF-conditioned medium effect on ampullary cancer cells

To investigate the influence of CAF on the AMPAC cancer cell proteome, we performed quantitative shotgun proteomics of AMPAC cells treated with CAF-CM versus controls under standard culture conditions, respectively. Quantitative proteome comparison was performed for cell lysates of all five AMPAC cell lines. Stable isotope labeling with either ^12^COH_2_ formaldehyde (light) or ^13^COD_2_ formaldehyde (heavy) was used for relative quantitation.

**Fig. 7:**
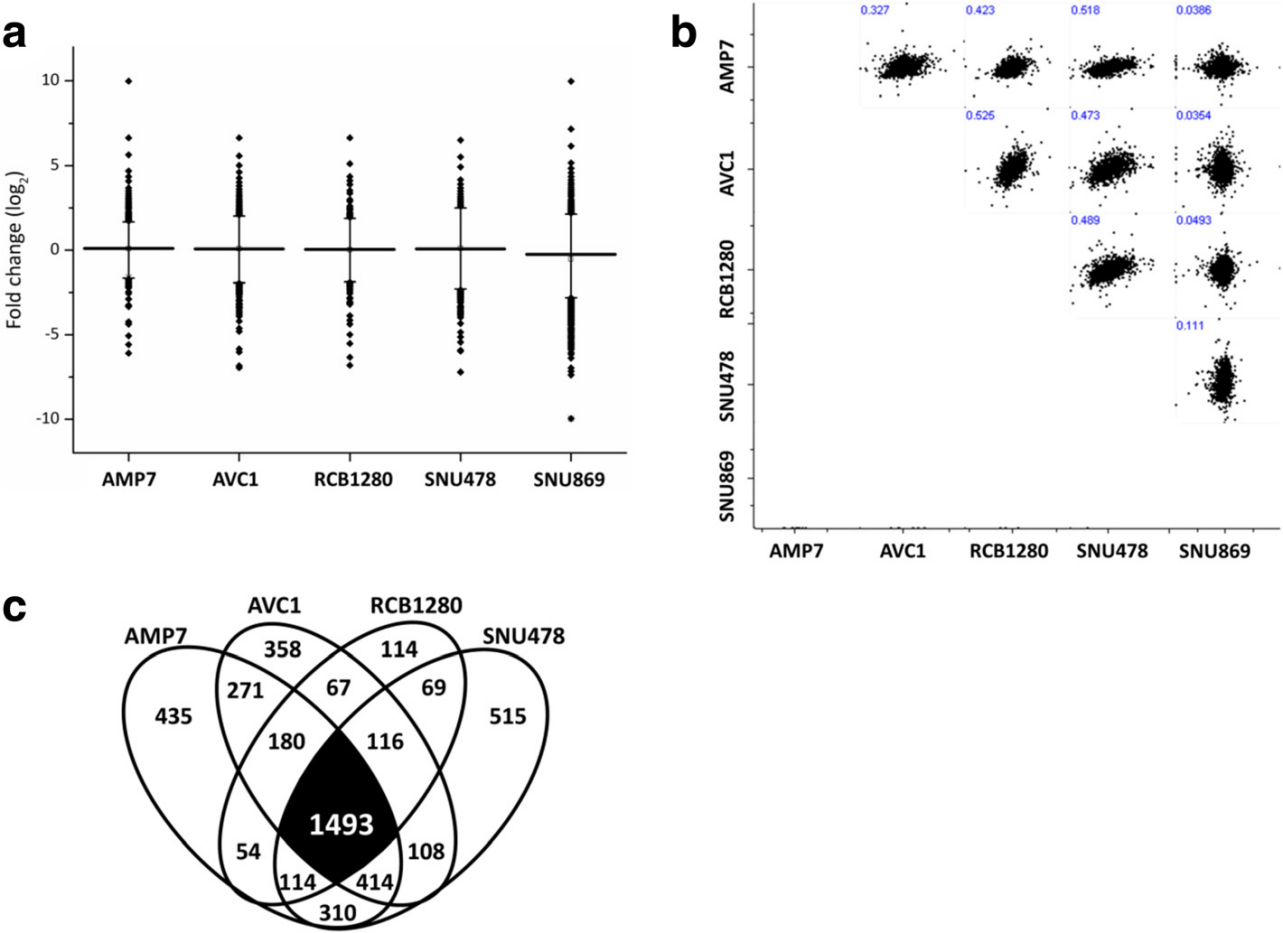
Distribution of identified proteins in AMPAC cells cultivated in CAF conditioned medium. **a** Geometric mean (horizontal bar) of fold change values (log_2_ of CAF-treated/control) of proteins from each AMPAC cell line comparing the CAF conditioned medium treatment or control. **b** Fold change correlation of all quantified proteins between different AMPAC cell lines treated with CAF-conditioned medium. **c** Venn diagram showing the overlap of proteins identified in individual AMPAC cell line upon cultivation with CAF conditioned medium or control

LC-MS/MS analysis identified 3 258 proteins in MDA-AMP7, 3 007 proteins in AVC1, 2 207 proteins in RCB1280, 3 139 proteins in SNU487 and 4 376 proteins in SNU869. The fold-change values (Fc-values, log_2_ of CAF-treated/untreated ratio) show a near-normal distribution for all five replicates comparing CAF-conditioned medium treated and non-treated cells (Fig. 7a). These findings indicate the majority of proteins not being affected in abundance upon cultivation in CAF-conditioned medium. Furthermore, there is a moderate level of correlation between the Fc-values of the MDA-AMP7, AVC1, RCB1280 and SNU487 (Fig. 7b) indicating a comparable reaction in proteome composition of these cell lines to cultivation in CAF-conditioned medium. Consequently, an overlap of 1 493 proteins was identified among these four AMPAC cell lines (Fig. 7c). Partially incomplete overlap of proteome coverage is an intrinsic characteristic of mass spectrometry-based proteomics [45]. On the other hand, the Fc-values of the SNU869 cell line lack appreciable correlation to any of the other cell lines; suggesting a different rearrangement of proteome composition upon cultivation in CAF-conditioned medium.

Proteins were considered as being differentially abundant upon cultivation in CAF-conditioned medium if the corresponding Fc-value (log_2_ of heavy:light ratio) is either greater than 0.58 or less than -0.58, corresponding to a change in abundance exceeding 50 %. Mass spectrometry analysis showed that 1 184 proteins were significantly affected in MDA-AMP7; 1 323 in AVC1; 876 in RCB1280; 1 603 in SNU487; and 745 in SNU869. A total of 345 proteins were commonly affected in these four AMPAC cell lines (MDA-AMP7, AVC1, RCB1280 and SNU487) (*p* < 0.10, two-tailed *t*-test). Further 87 proteins were commonly affected in three cell lines with adequate correlation (*p* < 0.10, two-tailed *t*-test). Collectively, a total of 432 proteins were differentially affected more than two-fold in at least three cell lines (n ≥ 3); 208 up-regulated (log_2_ ratio > 0.58, *p* < 0.10, two-tailed *t*-test), and 224 down-regulated (log_2_ ratio > 0.58, *p* < 0.10, two-tailed *t-*test). Additional data is listed in Additional file 1: Tables S1 and S2.

### Ingenuity pathway analysis of CAF-conditioned medium effects on ampullary cancer cells

**Fig. 8:**
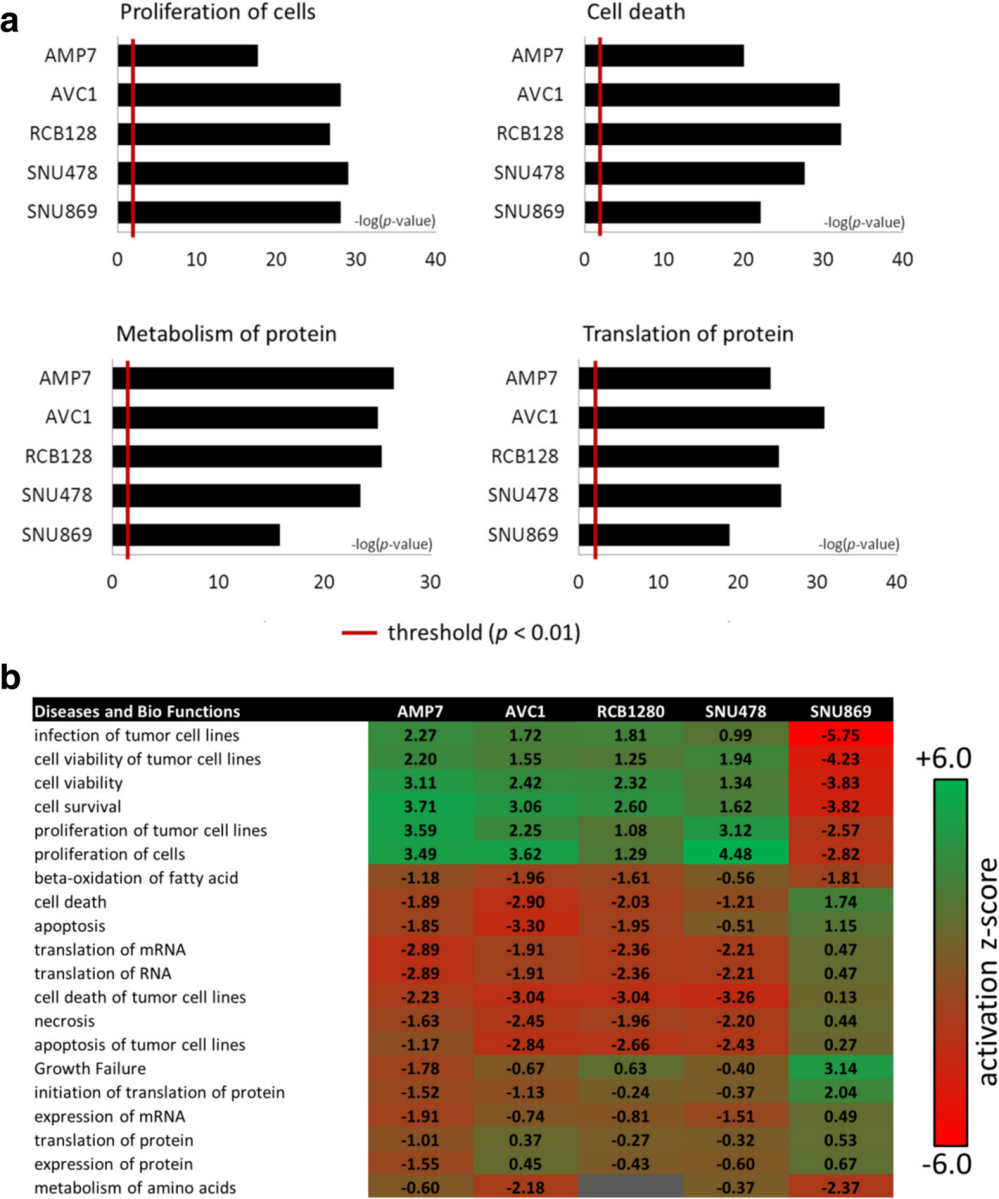
Bioinformatics analysis of quantified proteins by ingenuity pathway analysis. **a** Patho-physiological pathways and processes of proteins from AMPAC cell lines following treatment with CAF-conditioned medium or control. The threshold denotes statistical significance at *p* < 0.01. **b** Hierarchical clustering based on activation z-score, showing likelihood of regulating proteins based on a statistically significant pattern match of up- and down-regulation of biological processes

Ingenuity pathway analysis was used to elucidate biological themes being induced or repressed in AMPAC cells upon cultivation in CAF-conditioned medium. Comparison of the expression of all affected proteins (with pre-defined log_2_ cut-off value at ± 0.5) revealed that several pathways including cellular proliferation and metabolism, cell death, and protein synthesis were significantly affected (*p*-value < 0.01) (Fig. 8a). Hierarchical clustering of all proteins identified in each AMPAC cell lines, based on activation *z*-score, showed similar likelihood of regulating proteins (based on a statistically significant pattern match of up- and down-regulation of biological processes) between the mesenchymal-like cell lines MDA-AMP7, AVC1, RCB1280 and the pancreatobiliary-like SNU487. In contrast, the more intestinal SNU869 showed regulation in the opposite manner in many identified biological themes.

**Fig. 9:**
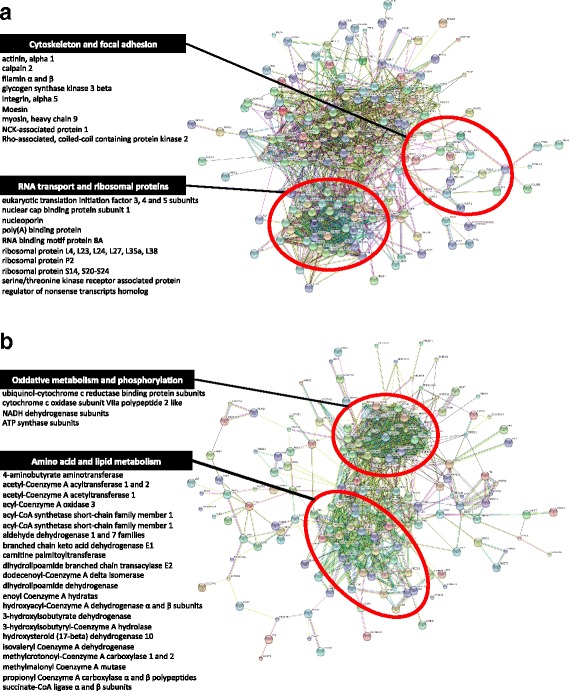
Network visualization of differentially regulated proteins by STRING in AMPAC cells upon cultivation with CAF conditioned medium. Clustering of proteins were based on KEGG pathway for (**a**) up-regulated proteins in at least 3 cell lines (Fc > 0.58, *p* ≤ 0.1), and (**b**) down-regulated proteins in at least 3 cell lines (Fc < -0.58, *p* ≤ 0.1)

The twenty most relevant biological themes affecting tumorigenesis and cancer progression of AMPAC cells upon the treatment with fibroblast conditioned medium are illustrated in Fig. 8b. Some of these biological processes include (a) positive regulation in cellular growth and proliferation, as well as infection and proliferation of tumor cell lines; and (b) negative regulation in tumor cell death and apoptosis. Many of these biological networks and pathways were also identified in Gene Ontology (GO) enrichment analysis. Gene Ontology (GO) enrichment analysis within the AMPAC subset of four (mesenchymal- and pancreatobiliary-like) cell lines (MDA-AMP7, AVC1, RCB1280 and SNU487) showed that 208 up-regulated proteins were mainly enriched in mRNA metabolism, intracellular transport and activation of the cytoskeleton (Table 5). STRING protein-protein interactions and KEGG pathway enrichment analyses showed the majority of these proteins being involved in cellular motility, RNA transport and organization of ribosomal proteins (Fig. 9a). For SNU869, a total of 432 up-regulated proteins were mostly enriched in membrane and cellular component organization, as well as in metabolic processes (Table 6). For down-regulated proteins within the same subset of the four cell lines, GO enrichment analysis showed that the 224 proteins were mainly involved in metabolic processes and phosphorylation (Table 5). STRING analysis and KEGG pathway enrichment analyses grouped these proteins into two main clusters; oxidative metabolism and phosphorylation, and amino acid and lipid metabolism (Fig. 9b). On contrary, 314 proteins were down-regulated in SNU869, which mainly involved in protein translation and peptide biosynthetic processes (Table 6).

**Table 5:** Gene Ontology (GO) functional enrichment analysis regulated proteins in ampullary cancer cells MDA-AMP7, AVC1, RCB1280 and SNU487

GO terms of up-regulated proteins	Count	FDR
GO:0065007 Biological regulation	93	1.41E-02
GO:0071840 Cellular component organization or biogenesis	67	2.08E-06
GO:0051179 Localization	54	5.18E-04
GO:0016071 mRNA metabolic process	42	9.87E-23
GO:0032268 Regulation of cellular protein metabolic process	44	5.73E-11
GO:0050896 Response to stimulus	75	1.09E-02
GO terms of down-regulated proteins	Count	FDR
GO:0008152 Metabolic process	98	9.97E-04
GO:0009987 Cellular process	119	3.15E-03
GO:0044237 Cellular metabolic process	113	4.87E-11
GO:0009117 Nucleotide metabolic process	37	2.96E-12
GO:0016310 Phosphorylation	22	2.60E-03
GO:0006629 Lipid metabolic process	24	5.66E-04

Enrichment is analyzed using all differentially expressed proteins with fold-change of greater than 50 % in MDA-AMP7, AVC1, RCB1280 and SNU487 cells cultivated in CAF-conditioned medium versus non-treated control cells (log_2_ ratio of > 0.58, *p*-value < 0.1, two-tailed *t*-test, FDR < 0.05)

*Abbreviations*: *CAF* cancer associated fibroblast, *FDR* false discovery rate

**Table 6:** Gene Ontology (GO) functional enrichment analysis regulated proteins in ampullary cancer cells SNU869

GO terms of up-regulated proteins	Count	FDR
GO:0071840 Cellular component organization or biogenesis	148	4.19E-07
GO:0061024 Membrane organization	47	7.99E-07
GO:0016043 Cellular component organization	143	7.99E-07
GO:0044238 Primary metabolic process	216	2.47E-06
GO:0044237 Cellular metabolic process	213	2.83E-06
GO:0045184 Establishment of protein localization	58	3.67E-06
GO terms of down-regulated proteins	count	FDR
GO0043043: Peptide biosynthetic process	30	1.18E-10
GO:0006412 Translation	29	1.18E-10
GO:0043604 Amide biosynthetic process	31	5.81E-10
GO:1901564 Organonitrogen compound metabolic process	61	1.24E-09
GO:0007005 Mitochondrion organization	29	3.54E-09
GO:0006518 Peptide metabolic process	30	5.57E-09

Enrichment is analyzed using all differentially expressed proteins with fold-change of greater than 50 % SNU869 cells cultivated in CAF-conditioned medium versus non-treated control cells (log_2_ ratio of > 0.58, *p*-value < 0.1, two-tailed *t*-test, FDR < 0.05)

Abbreviations: *CAF* cancer associated fibroblast, *FDR* false discovery rate

## Discussion

AMPAC is a relatively rare tumor comprising about 15–20 % of all adenocarcinomas resected by pancreatoduodenectomy [6, 46–48]. The most important prognostic factors reported in the literature include surgical margin, lymph node metastasis and intestinal differentiation. The latter has been highlighted as a decisive parameter for clinical prognosis. Its tumor biology is less aggressive and postulated to be more related to colorectal cancer [49]. Therefore, suggested analogous therapy such as liver metastasis resection as well as chemotherapy regimens used for colorectal cancer might be indicative for intestinal AMPAC [50–52]. Despite the growing number of cell lines and increasing relevance of cell line based high-throughput screening methods [21, 22], establishment of ampullary cancer cell lines has only been limited [23–26]. Moreover, *in vitro* experimental data on AMPAC is absent. Hence, our aim is to establish an experimental platform for the study of AMPAC biology.

Clinical data from patients treated at our center confirmed that intestinal subtype is a main prognostic factor and was significantly associated with KRT 20 and CDX2 expression and KRT7 negativity. A novel aspect was a significant association between intestinal differentiation and reduced features of EMT (tumor budding, ZEB1 and E-Cadherin expression). We were able to obtain and grow one cell line from each of the five reported patients (a total five AMPAC cell lines) under the same standard culture conditions. Basic characterization on the basis of markers for subtype and EMT revealed remarkable heterogeneity among these cell lines. However, hierarchical clustering analyses combining tumors and cell lines based on expression pattern suggested three main pattern: mesenchymal-like, PB, and INT differentiation. Interestingly, these observations also reflect the origin of the cell lines: two out of the three mesenchymal-like cell lines were isolated from metastases, where theoretically increased EMT features would be postulated. The AVC1 cells were derived from a moderately differentiated primary tumor, but displayed a loss of differentiation markers and partial EMT. The pancreatobiliary-like cell line SNU478 was derived from an AMPAC with signet ring cell features, which is a rare and aggressive tumor [53]. Loss of E-Cadherin by germline mutation is associated with diffuse gastric adenocarcinoma with signet ring cells [54]. Similarly, SNU478 has been shown to have the CDH1 gene repressed by DNA methylation [24] and displayed similarly high invasiveness as the mesenchymal-like cells.

Among the five cell lines, only one intestinal-like cell line (SNU869) was found, which is not surprising given that it was previously suggested that only the most aggressive tumor cells can be grown as cell lines in standard culture conditions [55]. Notably, SNU869 is the only cell line reported to be derived from a well-differentiated primary AMPAC tumor. SNU869 cells displayed a markedly reduced growth and invasiveness compared to the other cell lines, as would be expected from clinical correlation. Interestingly, this intestinal-like cell line was not sensitive to low concentration of Gemcitabine, a drug often used to treat ampullary cancer. This is consistent with current clinical reports suggesting poor response or even reduced survival with Gemcitabine-based chemotherapy in intestinal type AMPAC [44, 56, 57].

Cancer associated fibroblasts (CAF) are known to promote tumorigenesis, invasion and metastasis [58, 59]. Additionally, the prognostic value of CAF’s immunohistochemical markers as well as stromal gene expression profiles have been previously reported [60–63]. However there is currently no standardized grading system to look at stromal CAF activity [63]. Here, we adopted a simple stromal grading system relying on morphological features of CAF [28] and noted increased CAF activation and EMT features in non-intestinal AMPAC. Therefore we tested the hypothesis in which CAF could enhance growth and induce EMT and a phenotypic shift in AMPAC cells through CAF secreted factors.

Treatment of AMPAC cells with CAF-CM from CAF isolated from a human AMPAC had several effects. In general, cellular growth and invasion were increased, ZEB1 expression increased and/or E-Cadherin expression decreased in the cell lines where these genes were expressed. With regard to AMPAC subtype marker expression, decrease of the intestinal markers KRT20 and CDX2 were observed. The cell line derived from signet ring cell AMPAC (SNU478) however displayed decreased cellular invasion, and increased CDX2 and KRT7 genes expression. The undifferentiated MDA-AMP7 cell line showed a slight decrease compared to the very high ZEB1 baseline expression. These findings finding in which CAF-conditioned medium enhanced AMPAC cell growth and EMT features while suppressing intestinal markers and increasing KRT7 are well correlated with clinical data from histopathology and patient survival. It is also known that some cancer cell lines display a loss of differentiation markers and E-Cadherin in standard culture [62, 64, 65]. However these features were not observed in the five cell lines.

Proteomic analysis revealed parallel regulation shifts in the mesenchymal- and pancreatobiliary-like cell lines. Pathophysiological pathways of cellular proliferation and survival, activation of the cytoskeleton and intracellular transport were up-regulated, whereas pathways of cell apoptosis, protein translation, oxidative, amino acid and lipid metabolism were down-regulated. These findings suggest a supportive role of CAF in AMPAC cell survival, proliferation and motility. Of note, the intestinal-like cell line SNU869 showed many of these regulation processes to respond in an opposite manner, suggesting CAF response to be differentiation-dependent in AMPAC. In addition, KRT20, CDX 2, KRT 7, ZEB 1 and CDH 1 were not consistently identified in all the cell lines (Additional file 1: Table S1, complete mass spectrometry data available via ProteomeXchange with identifier PXD002657). This is not surprising, as undersampling and partially incomplete overlap of proteome coverage is an intrinsic characteristic of mass-spectrometry based studies [45].

A major drawback of this study is that classification of cell lines relies on in vitro parameters and correlation with an independent clinical dataset from tumors. However, no information on the subtype of the tumors that the cell lines were derived from, nor patient follow-up, is available for validation of conclusions drawn. Up to now, this remains a general problem of virtually all standard cancer cell lines. Even the most advanced large scale cell line panel characterization studies have not been validated using this approach [19–22]. Nevertheless, these studies suggest that cell lines do reflect important aspects of the clinical tumor biology, like marker expression and drug sensitivity.

## Conclusions

On the basis of clustering for EMT features and subtype marker expression, most of the available AMPAC cell lines seem to reflect a poorly differentiated pancreatobiliary or mesenchymal-like phenotype, which is consistent with their state of origin from moderate to poorly differentiated primary tumors or metastases. Only SNU869, which is derived from a well-differentiated primary tumor, displays an intestinal phenotype and characteristically segregated from the other cell lines specifically in basic marker expression, drug sensitivity as well as proteomic CAF-response. Based on these findings, we therefore suggest that SNU869 is presently the most appropriate cell-based model for intestinal-like AMPAC, while others seem to reflect aggressive and metastatic AMPAC subtypes.

## Additional file

Additional file 1: Table S1. List of up-regulated proteins identified from ampullary cancer cells cultivated with CAF conditioned medium. **Table S2.** List of down-regulated proteins identified from ampullary cancer cells treated with CAF conditioned medium. (DOCX 94 kb)

## Abbreviations

AMPAC: ampullary adenocarcinoma
AT: atypical
ATCC: American TYPE CULTURE COLLECTION
CAF: cancer associated fibroblast
CDH1: E-cadherin
CDX2: caudal type homeobox 2
CM: conditioned medium
D: differentiated
DMEM: Dulbecco’s modified Eagle’s medium
DMSO: dimethyl sulfoxide
DNA: deoxyribonucleic acid
ECad: E-cadherin
EMT: epithelial-mesenchymal transition
G: histopathologic tumor grade
GO: gene ontology
H&E: hematoxylin-eosin
HCL: hierarchical clustering
INT: intestinal
KEGG: Kyoto Encyclopedia of genes and genomes
KRT20: cytokeratin 20
KRT7: Cytokeratin 7
L: LYMPHANGIOSIS
LNR: lymph node ratio
MES: mesenchymal-like
mRNA: messenger ribonucleic acid
MTT: 3-(4,5-dimethylthiazol-2-yl)-2,5-diphenyltetrazolium bromide
PanCK: Pan-Cytokeratin
PB: pancreatobiliary
PCR: polymerase chain reaction
PDAC: pancreatic ductal adenocarcinoma
POOR: poorly differentiated
R: resection margin status
STRING: Search Tool for the Retrieval of Interacting Genes/Proteins
T: histopathologic tumor stage
TNM: tumor-lymph nodes-metastasis classification
VIM: vimentin
ZEB1: zinc finger E-box binding homeobox 1
