# Supplementary material for: Characterization of various cell lines from different ampullary cancer subtypes and cancer associated fibroblast-mediated responses
Source: BMC Cancer. 2016 Mar 8;16:195. doi: 10.1186/s12885-016-2193-5 (PMC4782372; doi:10.1186/s12885-016-2193-5)
Supplement: Additional file 1: Table S1. — List of up-regulated proteins identified from ampullary cancer cells cultivated with CAF conditioned medium. Table S2. List of down-regulated proteins identified from ampullary cancer cells treated with CAF conditioned medium. (DOCX 94 kb) [file 12885_2016_2193_MOESM1_ESM.docx]

**Additional file 1**

**Table S1: List of up-regulated proteins identified from ampullary cancer cells cultivated with CAF conditioned medium.**

|  | **Fold change (log_2_ of CAF medium treated/control)** | | | | | | |
| --- | --- | --- | --- | --- | --- | --- | --- |
| **Protein Name** | **AMP7** | **AVC1** | **RCB1280** | **SNU478** | **Fc mean** | **Fc error** | **p-value** |
| 14-3-3 protein theta; | 0.10 | 0.62 | 0.99 | 1.32 | 0.76 | 0.52 | 0.085 |
| 26S protease regulatory subunit 4; | 0.50 | 1.90 | 0.29 | 1.40 | 1.02 | 0.76 | 0.091 |
| 26S protease regulatory subunit 7; | 0.44 | 1.24 | 0.50 | 0.88 | 0.77 | 0.37 | 0.036 |
| 26S proteasome non-ATPase regulatory subunit 1; | 0.21 | 0.99 | 0.52 | 1.24 | 0.74 | 0.46 | 0.068 |
| 26S proteasome non-ATPase regulatory subunit 11; | 0.67 | 0.90 | 0.35 | 0.58 | 0.63 | 0.23 | 0.019 |
| 26S proteasome non-ATPase regulatory subunit 14; | 0.67 | 0.62 | 0.50 | 0.76 | 0.64 | 0.11 | 0.002 |
| 26S proteasome non-ATPase regulatory subunit 2; | 0.65 | 1.79 | 1.01 | 0.79 | 1.06 | 0.51 | 0.032 |
| 28 kDa heat- and acid-stable phosphoprotein; | 0.18 | 1.24 | 0.96 | 1.13 | 0.88 | 0.48 | 0.047 |
| 40S ribosomal protein S10; | 0.16 | 0.53 | 0.80 | 0.94 | 0.61 | 0.34 | 0.058 |
| 40S ribosomal protein S14; | 0.16 | 0.62 | 1.10 | 1.06 | 0.74 | 0.44 | 0.062 |
| 40S ribosomal protein S17; | 0.10 | 0.84 | 0.54 | 0.85 | 0.58 | 0.35 | 0.069 |
| 40S ribosomal protein S2; | 0.46 | 0.58 | 0.29 | 1.24 | 0.64 | 0.42 | 0.078 |
| 40S ribosomal protein S20; | 0.46 | 0.87 | 0.62 | 0.63 | 0.65 | 0.17 | 0.007 |
| 40S ribosomal protein S21; | 0.41 | 0.96 | 0.31 | 1.40 | 0.77 | 0.51 | 0.076 |
| 40S ribosomal protein S23; | 0.41 | 0.62 | 0.60 | 1.45 | 0.77 | 0.46 | 0.061 |
| 40S ribosomal protein S24; | 0.63 | 0.41 | 0.58 | 1.49 | 0.78 | 0.48 | 0.066 |
| 40S ribosomal protein S27; | 0.00 | 0.77 | 0.99 | 1.21 | 0.74 | 0.53 | 0.090 |
| 40S ribosomal protein SA; | 0.50 | 0.47 | 0.56 | 0.94 | 0.62 | 0.22 | 0.017 |
| 60S acidic ribosomal protein P0; | 0.28 | 0.82 | 0.38 | 0.85 | 0.58 | 0.29 | 0.045 |
| 60S acidic ribosomal protein P2; | 0.39 | 0.67 | 0.48 | 0.94 | 0.62 | 0.24 | 0.023 |
| 60S ribosomal protein L23; | 0.50 | 0.30 | 1.33 | 1.10 | 0.81 | 0.49 | 0.061 |
| 60S ribosomal protein L24; | 0.31 | 0.41 | 0.50 | 1.24 | 0.62 | 0.42 | 0.091 |
| 60S ribosomal protein L27; | 0.18 | 0.90 | 0.93 | 1.06 | 0.77 | 0.40 | 0.043 |
| 60S ribosomal protein L35a; | 0.12 | 1.28 | 0.64 | 1.74 | 0.95 | 0.71 | 0.096 |
| 60S ribosomal protein L38; | 0.12 | 0.82 | 1.19 | 1.36 | 0.87 | 0.55 | 0.066 |
| 60S ribosomal protein L4; | 0.30 | 0.47 | 0.54 | 1.06 | 0.59 | 0.33 | 0.056 |
| 60S ribosomal protein L7; | 0.44 | 0.58 | 0.21 | 1.24 | 0.62 | 0.44 | 0.098 |
| 6-phosphofructokinase type C; | 0.54 | 0.62 | 1.04 | 1.91 | 1.03 | 0.63 | 0.059 |
| 6-phosphofructokinase, muscle type; | 1.09 | 0.03 | 0.73 | 1.03 | 0.72 | 0.49 | 0.082 |
| Activating signal cointegrator 1 complex subunit 3; | 1.21 | 1.04 |  | 0.94 | 1.06 | 0.54 | 0.091 |
| Acyl-CoA-binding protein; | -0.03 | 0.90 | 0.99 | 1.21 | 0.77 | 0.55 | 0.091 |
| Adenosylhomocysteinase; | 0.44 | 0.77 | 0.80 | 1.21 | 0.81 | 0.32 | 0.020 |
| Adenylosuccinate synthetase isozyme 2; | 0.58 | 1.43 | 0.83 | 1.85 | 1.17 | 0.58 | 0.033 |
| Alpha-actinin-1; | 0.65 | 1.11 | 0.35 | 1.06 | 0.79 | 0.36 | 0.030 |
| Alpha-taxilin; |  | 1.39 | 1.01 | 1.45 | 1.28 | 0.67 | 0.092 |
| Asparagine synthetase [glutamine-hydrolyzing]; | 0.72 | 1.43 | 1.40 | 2.10 | 1.41 | 0.56 | 0.018 |
| Ataxin-2-like protein; | 0.87 | 0.87 | 0.96 | 1.06 | 0.94 | 0.09 | 0.000 |
| ATP-dependent DNA helicase Q1; | 0.37 | 0.24 | 0.78 | 0.97 | 0.59 | 0.34 | 0.062 |
| Basic leucine zipper and W2 domain-containing protein 1; | 0.92 | 1.04 | 0.60 | 1.32 | 0.97 | 0.30 | 0.010 |
| C-1-tetrahydrofolate synthase, cytoplasmic; | 0.28 | 0.43 | 0.56 | 1.13 | 0.60 | 0.37 | 0.072 |
| Calcyclin-binding protein; | 0.94 | 1.79 | 0.64 | 1.49 | 1.22 | 0.52 | 0.023 |
| Calpain-2 catalytic subunit; | 0.94 | 0.08 | 0.99 | 1.06 | 0.77 | 0.46 | 0.061 |
| Calponin-2; | 0.33 | 0.79 | 0.62 | 1.10 | 0.71 | 0.32 | 0.031 |
| Cdc42 effector protein 4; |  | 2.08 | 2.16 | 1.54 | 1.93 | 1.00 | 0.087 |
| Cellular nucleic acid-binding protein; | 0.33 | 0.26 | 1.01 | 0.76 | 0.59 | 0.36 | 0.069 |
| Chloride intracellular channel protein 1; | 0.92 | 0.24 | 0.73 | 0.82 | 0.68 | 0.30 | 0.031 |
| C-Jun-amino-terminal kinase-interacting protein 4; | 0.56 | 0.56 | 0.50 | 1.40 | 0.76 | 0.43 | 0.055 |
| CTP synthase 1; | 0.39 | 0.74 | 0.93 | 1.24 | 0.83 | 0.36 | 0.026 |
| CUB domain-containing protein 1; | 1.03 | 1.43 | 0.62 | 0.26 | 0.84 | 0.51 | 0.061 |
| Cysteine and histidine-rich domain-containing protein 1; | 0.56 | 0.90 | 0.54 | 2.17 | 1.04 | 0.77 | 0.090 |
| Cytosolic Fe-S cluster assembly factor NUBP2; | 0.81 |  | 0.62 | 0.66 | 0.70 | 0.36 | 0.100 |
| D-3-phosphoglycerate dehydrogenase; | 0.54 | 0.93 | 0.80 | 1.00 | 0.82 | 0.20 | 0.006 |
| DNA (cytosine-5)-methyltransferase 1; | 1.24 | 1.64 |  | 1.40 | 1.43 | 0.73 | 0.087 |
| DNA mismatch repair protein Msh6; | 1.03 | 0.43 | 1.40 | 1.00 | 0.97 | 0.40 | 0.022 |
| DNA replication licensing factor MCM5; | 0.87 | 1.79 | 2.60 | 0.54 | 1.45 | 0.93 | 0.062 |
| DNA replication licensing factor MCM6; | 1.00 | 2.35 | 1.88 | 1.79 | 1.76 | 0.56 | 0.010 |
| DNA replication licensing factor MCM7; | 1.35 | 1.60 |  | 1.68 | 1.54 | 0.78 | 0.085 |
| DNA topoisomerase 1; | 1.06 | 1.60 | 0.80 | 1.63 | 1.27 | 0.41 | 0.010 |
| E3 ubiquitin-protein ligase UBR4; | 0.67 | 0.72 | 2.10 | 1.49 | 1.25 | 0.68 | 0.043 |
| Elongation factor 1-gamma; | 0.37 | 1.01 | 0.52 | 1.17 | 0.77 | 0.38 | 0.039 |
| Elongation factor 2; | 0.56 | 0.62 | 0.66 | 1.54 | 0.85 | 0.47 | 0.048 |
| Enhancer of mRNA-decapping protein 4; | 0.46 | 0.65 | 0.80 | 0.71 | 0.66 | 0.14 | 0.004 |
| Eukaryotic initiation factor 4A-I; | 0.79 | 1.31 | 0.80 | 1.59 | 1.12 | 0.40 | 0.014 |
| Eukaryotic peptide chain release factor GTP-binding subunit ERF3A; | 0.61 | 0.62 | 0.60 | 1.45 | 0.82 | 0.42 | 0.041 |
| Eukaryotic peptide chain release factor subunit 1; | 0.35 | 1.24 | 0.71 | 0.97 | 0.82 | 0.38 | 0.032 |
| Eukaryotic translation initiation factor 3 subunit A; | 0.54 | 0.19 | 0.83 | 1.00 | 0.64 | 0.36 | 0.054 |
| Eukaryotic translation initiation factor 3 subunit B; | 0.61 | 0.72 | 0.88 | 1.59 | 0.95 | 0.44 | 0.030 |
| Eukaryotic translation initiation factor 3 subunit C; | 0.65 | 0.37 | 0.76 | 1.59 | 0.84 | 0.52 | 0.065 |
| Eukaryotic translation initiation factor 3 subunit I; | 1.03 | 0.67 | 0.29 | 2.10 | 1.02 | 0.78 | 0.097 |
| Eukaryotic translation initiation factor 3 subunit L; | 0.81 | 1.24 |  | 1.21 | 1.09 | 0.58 | 0.097 |
| Eukaryotic translation initiation factor 4 gamma 1; | 0.67 | 0.53 | 0.93 | 1.74 | 0.97 | 0.54 | 0.048 |
| Eukaryotic translation initiation factor 4 gamma 2; | 0.97 | 0.93 | 0.50 | 1.49 | 0.97 | 0.41 | 0.023 |
| Eukaryotic translation initiation factor 4B; | 0.26 | 0.60 | 0.83 | 1.32 | 0.75 | 0.44 | 0.059 |
| Eukaryotic translation initiation factor 4H; | 0.65 | 1.01 | 0.91 | 1.68 | 1.06 | 0.44 | 0.022 |
| Eukaryotic translation initiation factor 5; | 0.61 | 1.43 | 1.10 | 1.36 | 1.13 | 0.37 | 0.012 |
| Eukaryotic translation initiation factor 5B; | 0.07 | 1.21 | 0.80 | 1.06 | 0.79 | 0.51 | 0.072 |
| Eukaryotic translation initiation factor 6; | 0.61 | 0.14 | 0.73 | 0.85 | 0.58 | 0.31 | 0.051 |
| Exportin-2; | 0.56 | 1.17 | 1.51 | 0.94 | 1.05 | 0.40 | 0.018 |
| FAS-associated factor 1; | 1.59 | 1.79 |  | 2.40 | 1.93 | 1.02 | 0.090 |
| Fascin; | 1.28 | -0.01 | 0.96 | 1.68 | 0.98 | 0.72 | 0.092 |
| F-box-like/WD repeat-containing protein TBL1XR1; | 0.89 | 0.99 | 0.73 | 0.08 | 0.67 | 0.41 | 0.066 |
| Filamin-A; | 0.97 | 1.08 | 1.44 | 1.97 | 1.37 | 0.45 | 0.011 |
| Filamin-B; | 1.28 | 0.93 | 0.91 | 2.04 | 1.29 | 0.53 | 0.020 |
| Four and a half LIM domains protein 2; | 2.15 | 0.24 | 1.01 | 0.79 | 1.05 | 0.80 | 0.098 |
| General transcription factor IIF subunit 1; | 0.61 | 0.77 | 0.96 | 1.40 | 0.94 | 0.34 | 0.016 |
| Glutaredoxin-3; | 0.54 | 1.08 | 1.01 | 1.63 | 1.07 | 0.45 | 0.022 |
| Glycogen phosphorylase, liver form; | 0.97 | 1.64 | 0.35 | 1.06 | 1.01 | 0.53 | 0.041 |
| Glycogen synthase kinase-3 beta; | 0.42 | 0.58 | 0.50 | 1.32 | 0.71 | 0.42 | 0.060 |
| GMP synthase [glutamine-hydrolyzing]; | 0.94 | 1.01 | 0.99 |  | 0.98 | 0.49 | 0.089 |
| Heat shock 70 kDa protein 4; | 0.37 | 0.47 | 0.60 | 1.49 | 0.73 | 0.51 | 0.089 |
| Heat shock cognate 71 kDa protein; | 0.12 | 1.31 | 0.78 | 1.28 | 0.87 | 0.56 | 0.068 |
| Heat shock protein 105 kDa; | 1.00 | 1.21 | 0.56 | 2.40 | 1.29 | 0.79 | 0.055 |
| Heat shock protein HSP 90-alpha; | 0.61 | 1.11 | 0.62 | 1.79 | 1.03 | 0.56 | 0.043 |
| Heat shock protein HSP 90-beta; | 0.69 | 0.96 | 0.76 | 1.74 | 1.04 | 0.48 | 0.030 |
| Hemoglobin subunit alpha; | 1.35 | 1.24 | 1.78 | 0.51 | 1.22 | 0.53 | 0.024 |
| High mobility group protein B1; | 1.31 | 2.21 | 1.37 | 0.43 | 1.33 | 0.73 | 0.042 |
| High mobility group protein HMG-I/HMG-Y; |  | 1.17 | 1.83 | 1.24 | 1.41 | 0.77 | 0.096 |
| Hippocalcin-like protein 1; | 0.21 | 0.67 | 0.93 | 1.13 | 0.74 | 0.40 | 0.048 |
| Histone-binding protein RBBP4; | 0.33 | 0.93 | 0.28 | 1.00 | 0.64 | 0.38 | 0.066 |
| Histone-binding protein RBBP7; | 0.52 | 0.77 | 0.71 | 1.85 | 0.96 | 0.60 | 0.063 |
| Hsp90 co-chaperone Cdc37; | 0.50 | 1.35 | 0.38 | 1.54 | 0.94 | 0.59 | 0.063 |
| Hypoxanthine-guanine phosphoribosyltransferase; | 0.54 | 0.35 | 0.71 | 1.49 | 0.77 | 0.50 | 0.073 |
| Importin-5; | 0.74 | 0.90 | 0.62 | 1.54 | 0.95 | 0.41 | 0.025 |
| Importin-7; | 0.92 | 1.69 | 2.22 | 2.10 | 1.73 | 0.59 | 0.011 |
| Inosine-5'-monophosphate dehydrogenase 2; | 1.21 | 0.93 | 0.78 | 1.49 | 1.10 | 0.31 | 0.008 |
| Insulin-like growth factor 2 mRNA-binding protein 2; | 0.79 | 0.84 | 0.56 | 0.74 | 0.73 | 0.12 | 0.002 |
| Integrin alpha-5; | 1.31 | 0.72 | 0.93 | 0.76 | 0.93 | 0.27 | 0.008 |
| Intron-binding protein aquarius; | 0.61 | 2.02 | 0.91 | 0.47 | 1.00 | 0.70 | 0.081 |
| Isoleucine--tRNA ligase, cytoplasmic; | 0.13 | 0.53 | 0.91 | 1.10 | 0.67 | 0.43 | 0.076 |
| Kinesin-1 heavy chain; | 0.58 | 0.62 | 0.56 | 1.06 | 0.71 | 0.24 | 0.014 |
| Lactoylglutathione lyase; | 0.44 | 0.60 | 0.31 | 1.21 | 0.64 | 0.40 | 0.071 |
| Lamina-associated polypeptide 2, isoform alpha; | 0.06 | 0.74 | 1.37 | 1.06 | 0.81 | 0.56 | 0.084 |
| La-related protein 4; | 0.39 | 1.74 | 1.29 | 1.21 | 1.16 | 0.56 | 0.032 |
| Lupus La protein; | 0.06 | 0.93 | 0.73 | 1.03 | 0.69 | 0.44 | 0.072 |
| Lysine--tRNA ligase; | 0.26 | 0.51 | 0.93 | 1.10 | 0.70 | 0.38 | 0.051 |
| MAP7 domain-containing protein 1; |  | 1.79 | 1.23 | 1.49 | 1.50 | 0.79 | 0.090 |
| Metallothionein-1E; | 1.28 | 6.64 | 4.11 | 1.97 | 3.50 | 2.42 | 0.067 |
| Microtubule-actin cross-linking factor 1, isoforms 1/2/3/5; | 0.69 | 0.45 | 0.36 | 1.49 | 0.75 | 0.51 | 0.084 |
| Microtubule-associated protein 4; | 0.35 | 0.84 | 1.07 | 1.59 | 0.96 | 0.51 | 0.043 |
| Moesin; | 0.54 | 0.39 | 0.58 | 1.54 | 0.76 | 0.52 | 0.084 |
| Monofunctional C1-tetrahydrofolate synthase, mitochondrial; | 0.74 | 0.53 | 1.13 | 0.94 | 0.84 | 0.26 | 0.010 |
| Multifunctional protein ADE2; | 0.50 | 0.72 | 0.96 | 1.74 | 0.98 | 0.54 | 0.046 |
| Myosin-9; | 0.65 | 0.77 | 1.10 | 1.49 | 1.00 | 0.38 | 0.017 |
| Nck-associated protein 1; | 0.81 | 1.11 | 0.56 | 0.66 | 0.79 | 0.24 | 0.010 |
| Negative elongation factor E; | 0.41 | 1.17 | 1.78 | 0.66 | 1.01 | 0.61 | 0.057 |
| Neutral amino acid transporter B(0); | 0.61 | 1.24 | 0.58 | 0.43 | 0.72 | 0.36 | 0.040 |
| NFX1-type zinc finger-containing protein 1; | 2.15 | 3.15 |  | 3.04 | 2.78 | 1.46 | 0.086 |
| Nicotinamide phosphoribosyltransferase; | 0.84 | 0.84 | 0.88 | 0.79 | 0.84 | 0.04 | 0.000 |
| Nuclear autoantigenic sperm protein; | 0.97 | 1.14 | 2.44 | 2.04 | 1.65 | 0.71 | 0.022 |
| Nuclear cap-binding protein subunit 1; | 0.97 | 1.28 | 0.93 | 1.00 | 1.05 | 0.16 | 0.001 |
| Nuclear pore complex protein Nup50; | 0.44 | 0.99 | 1.04 | 0.85 | 0.83 | 0.27 | 0.012 |
| Nucleolin; | 0.25 | 1.28 | 0.73 | 1.32 | 0.90 | 0.51 | 0.051 |
| Nucleolysin TIAR; | 0.15 | 1.11 | 1.78 | 0.63 | 0.92 | 0.70 | 0.099 |
| Nucleosome assembly protein 1-like 1; | 0.41 | 0.90 | 1.48 | 2.17 | 1.24 | 0.76 | 0.057 |
| Oxysterol-binding protein 1; | 0.26 | 1.31 | 0.50 | 0.85 | 0.73 | 0.46 | 0.068 |
| Pachytene checkpoint protein 2 homolog; | 1.00 | 1.14 |  | 1.59 | 1.24 | 0.67 | 0.097 |
| PDZ and LIM domain protein 5; | 0.84 | 0.90 | 0.48 | 1.00 | 0.81 | 0.23 | 0.008 |
| PDZ and LIM domain protein 7; | 1.67 | 0.53 | 1.33 | 1.59 | 1.28 | 0.52 | 0.020 |
| Peptidyl-prolyl cis-trans isomerase FKBP4; | 0.39 | 1.47 | 1.33 | 2.04 | 1.31 | 0.68 | 0.038 |
| PEST proteolytic signal-containing nuclear protein; | 0.58 | 0.19 | 0.48 | 1.24 | 0.62 | 0.44 | 0.097 |
| Phenylalanine--tRNA ligase alpha subunit; | 0.20 | 0.93 | 0.66 | 1.24 | 0.76 | 0.44 | 0.057 |
| Phospholipase A-2-activating protein; | 0.61 | 0.62 | 0.62 | 0.88 | 0.68 | 0.13 | 0.003 |
| Plasminogen activator inhibitor 1 RNA-binding protein; | 0.09 | 0.90 | 1.04 | 1.21 | 0.81 | 0.50 | 0.063 |
| Poly(rC)-binding protein 2; | 0.54 | 0.93 | 0.46 | 0.94 | 0.72 | 0.25 | 0.016 |
| Polyadenylate-binding protein 1; | 0.61 | 0.72 | 0.31 | 1.13 | 0.69 | 0.34 | 0.039 |
| Pre-rRNA-processing protein TSR1 homolog; | 0.87 | 2.21 | 0.18 | 1.13 | 1.10 | 0.84 | 0.097 |
| Probable ATP-dependent RNA helicase DDX5; | 0.41 | 0.39 | 0.46 | 1.13 | 0.60 | 0.36 | 0.066 |
| Probable ATP-dependent RNA helicase DDX6; | 0.54 | 0.65 | 0.64 | 1.13 | 0.74 | 0.26 | 0.016 |
| Procollagen-lysine,2-oxoglutarate 5-dioxygenase 3; | 0.41 | 1.04 | 0.62 | 0.74 | 0.70 | 0.26 | 0.019 |
| Proliferating cell nuclear antigen; | 1.59 | 0.65 | 1.40 | 2.17 | 1.45 | 0.63 | 0.023 |
| Proliferation-associated protein 2G4; | 0.37 | 1.31 | 1.33 | 0.85 | 0.97 | 0.45 | 0.031 |
| Prostaglandin E synthase 3; | 0.54 | 0.69 | 0.83 | 1.54 | 0.90 | 0.44 | 0.036 |
| Proteasome activator complex subunit 3; | 0.63 | 1.64 | 1.10 | 1.91 | 1.32 | 0.57 | 0.023 |
| Protein AHNAK2; | 0.79 | 0.47 | 0.52 | 1.06 | 0.71 | 0.27 | 0.020 |
| Protein FAM50A; | 1.39 | 1.51 |  | 1.59 | 1.50 | 0.75 | 0.084 |
| Protein LSM14 homolog B; | 1.28 | 2.08 |  | 1.49 | 1.62 | 0.88 | 0.095 |
| Prothymosin alpha; | 0.44 | 1.79 | 1.48 | 1.54 | 1.31 | 0.60 | 0.027 |
| Putative pre-mRNA-splicing factor ATP-dependent RNA helicase DHX15; | 0.37 | 0.26 | 0.64 | 1.06 | 0.58 | 0.36 | 0.071 |
| Putative pre-mRNA-splicing factor ATP-dependent RNA helicase DHX16; | 1.97 | 1.35 | 0.58 | 3.68 | 1.90 | 1.32 | 0.072 |
| Rab11 family-interacting protein 5; | 0.89 | 1.35 | 0.33 | 0.63 | 0.80 | 0.43 | 0.047 |
| Ran-specific GTPase-activating protein; | 0.81 | 0.62 | 0.88 |  | 0.77 | 0.40 | 0.100 |
| Ras GTPase-activating protein-binding protein 1; | 0.69 | 0.87 | 0.50 | 1.32 | 0.85 | 0.35 | 0.023 |
| Regulator of nonsense transcripts 1; | 0.42 | 0.51 | 0.83 | 0.76 | 0.63 | 0.20 | 0.012 |
| Replication factor C subunit 5; | 1.18 | 0.72 | 0.96 | 1.59 | 1.11 | 0.37 | 0.012 |
| Rho GTPase-activating protein 18; | 0.30 | 1.47 | 1.26 | 1.24 | 1.07 | 0.52 | 0.034 |
| Rho-associated protein kinase 2; | 2.87 | 0.39 | 1.68 | 2.10 | 1.76 | 1.04 | 0.049 |
| Ribonuclease H2 subunit C; | 1.21 | 1.60 | 1.19 | 2.68 | 1.67 | 0.70 | 0.020 |
| Ribose-phosphate pyrophosphokinase 1; | 0.61 | 0.35 | 1.56 | 1.45 | 0.99 | 0.60 | 0.058 |
| RNA-binding protein 8A; | 0.44 | 0.39 | 0.88 | 0.76 | 0.62 | 0.24 | 0.022 |
| RuvB-like 2; | 0.25 | 1.04 | 0.88 | 1.24 | 0.85 | 0.43 | 0.038 |
| Septin-10; | 0.92 | 0.51 | 0.33 | 1.45 | 0.80 | 0.50 | 0.065 |
| Serine/threonine-protein phosphatase 6 regulatory subunit 3; | 0.63 | 0.45 | 0.29 | 1.10 | 0.62 | 0.35 | 0.058 |
| Serine-threonine kinase receptor-associated protein; | 0.46 | 1.17 | 1.23 | 1.91 | 1.19 | 0.59 | 0.034 |
| Sister chromatid cohesion protein PDS5 homolog A; | 0.52 | 0.56 | 1.01 | 0.40 | 0.62 | 0.27 | 0.029 |
| Spermine synthase; | 0.87 | 0.69 |  | 0.66 | 0.74 | 0.38 | 0.099 |
| SRSF protein kinase 2; | 0.97 |  | 1.01 | 1.49 | 1.16 | 0.62 | 0.098 |
| Stathmin; | 0.81 | 0.82 | 1.68 | 1.45 | 1.19 | 0.44 | 0.016 |
| STE20-like serine/threonine-protein kinase; | 0.65 | 0.41 | 1.04 | 0.94 | 0.76 | 0.29 | 0.019 |
| Stress-induced-phosphoprotein 1; | 0.41 | 1.01 | 1.07 | 1.36 | 0.96 | 0.40 | 0.022 |
| Structural maintenance of chromosomes protein 4; | 1.59 | 1.95 |  | 2.10 | 1.88 | 0.96 | 0.085 |
| T-complex protein 1 subunit alpha; | 0.58 | 0.62 | 0.46 | 1.00 | 0.67 | 0.23 | 0.016 |
| T-complex protein 1 subunit delta; | 0.56 | 0.74 | 0.58 | 0.94 | 0.71 | 0.18 | 0.006 |
| T-complex protein 1 subunit epsilon; | 0.25 | 0.69 | 0.52 | 0.97 | 0.61 | 0.30 | 0.042 |
| T-complex protein 1 subunit eta; | 0.94 | 0.58 | 0.52 | 0.94 | 0.75 | 0.23 | 0.010 |
| T-complex protein 1 subunit theta; | 0.63 | 0.43 | 0.35 | 1.40 | 0.70 | 0.48 | 0.085 |
| T-complex protein 1 subunit zeta; | 0.54 | 0.53 | 0.58 | 0.94 | 0.65 | 0.20 | 0.011 |
| Transcription factor BTF3; | 0.58 | 0.96 | 1.23 | 1.91 | 1.17 | 0.56 | 0.031 |
| Transferrin receptor protein 1; | 0.79 | 1.31 | 0.33 | 0.61 | 0.76 | 0.41 | 0.048 |
| Transitional endoplasmic reticulum ATPase; | 0.58 | 0.39 | 0.56 | 1.49 | 0.76 | 0.50 | 0.076 |
| Translational activator GCN1; | 0.87 | 0.45 | 0.54 | 0.97 | 0.71 | 0.25 | 0.016 |
| Transportin-3; | 1.21 | 0.90 | 0.96 | 0.58 | 0.91 | 0.26 | 0.008 |
| Tripeptidyl-peptidase 2; | 0.58 | 0.21 | 0.58 | 1.03 | 0.60 | 0.34 | 0.057 |
| tRNA (cytosine(34)-C(5))-methyltransferase; | 0.39 | 0.72 | 0.69 | 1.54 | 0.84 | 0.49 | 0.057 |
| Tropomyosin alpha-3 chain; | 0.67 | 0.96 | 0.29 | 1.10 | 0.76 | 0.36 | 0.034 |
| Tropomyosin alpha-4 chain; | 1.39 | 2.14 | 0.64 | 0.85 | 1.26 | 0.67 | 0.040 |
| Tubulin beta chain; | 0.69 | 0.43 | 1.40 | 1.40 | 0.98 | 0.50 | 0.038 |
| Tubulin beta-6 chain; | 0.89 | 0.49 | 0.83 | 1.06 | 0.82 | 0.24 | 0.009 |
| Tubulin gamma-1 chain; | 0.54 | 0.22 | 1.40 | 0.85 | 0.75 | 0.50 | 0.079 |
| Ubiquitin carboxyl-terminal hydrolase 7; | 0.28 | 1.43 | 0.58 | 0.79 | 0.77 | 0.49 | 0.069 |
| Ubiquitin fusion degradation protein 1 homolog; | 0.79 | 0.39 | 0.52 | 1.00 | 0.68 | 0.27 | 0.024 |
| Ubiquitin-associated protein 2-like; | 0.52 | 0.60 | 0.62 | 1.13 | 0.72 | 0.28 | 0.021 |
| Valine--tRNA ligase; | 0.35 | 0.77 | 0.58 | 0.85 | 0.64 | 0.22 | 0.016 |
| Vesicle transport protein GOT1B; | 0.89 | 0.90 |  | 1.00 | 0.93 | 0.47 | 0.090 |
| Vigilin; | 0.26 | 0.51 | 0.73 | 1.10 | 0.65 | 0.36 | 0.052 |
| WD repeat-containing protein 1; | 0.56 | 0.33 | 0.71 | 0.94 | 0.64 | 0.26 | 0.024 |
| WW domain-containing adapter protein with coiled-coil; |  | 1.28 | 1.56 | 1.32 | 1.39 | 0.70 | 0.086 |
| Y-box-binding protein 3; | 0.72 | 0.77 |  | 1.00 | 0.83 | 0.43 | 0.098 |
| Zyxin; | 0.87 | 0.79 | 0.73 | 1.24 | 0.91 | 0.23 | 0.006 |

Shown is a comprehensive listing of all proteins which are differentially up-regulated in minimum of three AMPAC cell lines (AMP7, AVC1, RCB1280 and SNU487) with fold change of at least 50% (log_2_ ratio of > 0.58, *p*-value < 0.1, two-tailed t-test).

**Table S2: List of down-regulated proteins identified from ampullary cancer cells treated with CAF conditioned medium.**

|  | **Fold change (log2 ratio of CAF treated medium/control)** | | | | | | |
| --- | --- | --- | --- | --- | --- | --- | --- |
| **Protein Name** | **AMP7** | **AVC1** | **RCB1280** | **SNU478** | **Fc mean** | **Fc error** | **p-value** |
| 15 kDa selenoprotein; | -1.45 | -1.05 | -1.00 | -1.03 | -1.13 | 0.21 | 0.001 |
| 2,4-dienoyl-CoA reductase, mitochondrial; | -0.65 | -1.11 | -1.20 | -1.06 | -1.01 | 0.24 | 0.003 |
| 28S ribosomal protein S36, mitochondrial; | -0.62 |  | -1.13 | -0.98 | -0.91 | 0.50 | 0.074 |
| 2-oxoisovalerate dehydrogenase subunit alpha, mitochondrial; | -1.10 | -1.49 |  | -1.35 | -1.31 | 0.68 | 0.069 |
| 39S ribosomal protein L15, mitochondrial; | -0.60 | -1.21 |  | -1.18 | -1.00 | 0.57 | 0.081 |
| 39S ribosomal protein L23, mitochondrial; | -0.61 | -1.03 | -0.91 | -0.06 | -0.65 | 0.43 | 0.041 |
| 39S ribosomal protein L4, mitochondrial; | -0.37 | -0.67 | -1.38 | -0.38 | -0.70 | 0.47 | 0.044 |
| 39S ribosomal protein L40, mitochondrial; |  | -0.77 | -1.00 | -0.90 | -0.89 | 0.45 | 0.064 |
| 39S ribosomal protein L49, mitochondrial; | -0.49 |  | -0.47 | -0.82 | -0.59 | 0.34 | 0.072 |
| 3-hydroxyacyl-CoA dehydrogenase type-2; | -1.10 | -0.56 | -0.65 | -0.14 | -0.61 | 0.39 | 0.037 |
| 3-hydroxyisobutyrate dehydrogenase, mitochondrial; | -0.60 | -1.85 | -1.37 | -1.14 | -1.24 | 0.52 | 0.014 |
| 3-hydroxyisobutyryl-CoA hydrolase, mitochondrial; | -0.69 | -0.42 |  | -0.76 | -0.62 | 0.34 | 0.069 |
| 3-ketoacyl-CoA thiolase, mitochondrial; | -1.34 | -1.47 | -0.59 | -0.87 | -1.07 | 0.41 | 0.011 |
| 3-ketoacyl-CoA thiolase, peroxisomal; | -0.91 | -0.74 | -0.79 | -0.44 | -0.72 | 0.20 | 0.004 |
| 3-oxoacyl-[acyl-carrier-protein] synthase, mitochondrial; | -1.17 | -1.71 | -0.98 | -1.14 | -1.25 | 0.32 | 0.003 |
| 4-aminobutyrate aminotransferase, mitochondrial; | -1.60 | -1.35 |  | -1.26 | -1.40 | 0.72 | 0.069 |
| Acetyl-CoA acetyltransferase, mitochondrial; | -1.17 | -0.47 | -0.87 | -0.12 | -0.66 | 0.46 | 0.047 |
| Acetyl-coenzyme A synthetase 2-like, mitochondrial; | -1.31 | -2.36 |  | -2.59 | -2.09 | 1.18 | 0.086 |
| Aconitate hydratase, mitochondrial; | -1.03 | -0.87 | -0.55 | -0.24 | -0.67 | 0.35 | 0.022 |
| Acyl-CoA synthetase family member 2, mitochondrial; | -1.63 | -2.52 |  | -2.02 | -2.06 | 1.09 | 0.076 |
| Acyl-CoA-binding domain-containing protein 5; | -2.22 | -2.32 |  | -1.27 | -1.94 | 1.08 | 0.082 |
| Adenylate kinase 2, mitochondrial; | -0.44 | -0.67 | -1.18 | -0.90 | -0.80 | 0.32 | 0.011 |
| Adenylate kinase 4, mitochondrial; | -1.17 | -1.14 |  | -0.68 | -1.00 | 0.55 | 0.075 |
| ADP-ribosylation factor-like protein 6-interacting protein 1; | 0.07 | -0.72 | -0.70 | -1.06 | -0.60 | 0.48 | 0.062 |
| AFG3-like protein 2; | -0.84 | -0.22 | -1.22 | -0.11 | -0.60 | 0.52 | 0.078 |
| Alanine--tRNA ligase, mitochondrial; | -0.38 | -0.83 | -0.39 | -0.80 | -0.60 | 0.25 | 0.011 |
| Aldehyde dehydrogenase X, mitochondrial; | -0.78 | -0.84 |  | -0.33 | -0.65 | 0.40 | 0.083 |
| All-trans-retinol 13,14-reductase; | -1.08 | -1.02 | -0.09 | -0.31 | -0.63 | 0.50 | 0.064 |
| Alpha-1,3/1,6-mannosyltransferase ALG2; | -0.49 | -1.28 | -0.29 | -0.89 | -0.74 | 0.44 | 0.032 |
| Alpha-aminoadipic semialdehyde dehydrogenase; | -0.70 | -0.65 | -0.90 | -0.66 | -0.73 | 0.12 | 0.001 |
| Amyloid beta A4 protein; | -1.04 |  | -1.25 | -0.65 | -0.98 | 0.55 | 0.077 |
| Antigen peptide transporter 1; | -0.54 |  | -0.47 | -0.87 | -0.63 | 0.36 | 0.074 |
| Apolipoprotein O; | -0.60 | -1.01 |  | -0.76 | -0.79 | 0.43 | 0.071 |
| ATP synthase F(0) complex subunit B1, mitochondrial; | -0.79 | -0.37 | -0.84 | -0.73 | -0.68 | 0.21 | 0.005 |
| ATP synthase subunit a; | -0.63 |  | -1.26 | -0.91 | -0.93 | 0.53 | 0.079 |
| ATP synthase subunit alpha, mitochondrial; | -0.68 | -0.37 | -0.94 | -0.54 | -0.63 | 0.24 | 0.009 |
| ATP synthase subunit d, mitochondrial; | -0.55 |  | -0.64 | -0.61 | -0.60 | 0.30 | 0.058 |
| ATP synthase subunit delta, mitochondrial; | -0.91 | -0.47 | -1.08 | -0.64 | -0.78 | 0.27 | 0.008 |
| ATP synthase subunit e, mitochondrial; | -0.74 | -0.50 | -1.04 | -0.54 | -0.71 | 0.25 | 0.007 |
| ATP synthase subunit epsilon, mitochondrial; | -0.23 | -1.35 | -1.29 | -0.78 | -0.91 | 0.52 | 0.031 |
| ATP synthase subunit f, mitochondrial; | -0.95 | -0.49 | -1.11 | -0.51 | -0.77 | 0.31 | 0.012 |
| ATP synthase subunit g, mitochondrial; | -0.79 | -0.40 | -0.63 | -0.53 | -0.59 | 0.16 | 0.004 |
| ATP synthase-coupling factor 6, mitochondrial; | -1.00 | -0.17 | -1.43 | -0.70 | -0.83 | 0.53 | 0.040 |
| ATPase family AAA domain-containing protein 1; | -1.10 | -0.31 | -0.81 | -0.21 | -0.61 | 0.42 | 0.045 |
| ATP-dependent RNA helicase SUPV3L1, mitochondrial; | -0.45 | -0.80 | -0.91 | -0.31 | -0.62 | 0.28 | 0.015 |
| Bcl-2-like protein 13; | -1.23 | -0.42 | -0.50 | -0.89 | -0.76 | 0.37 | 0.020 |
| Beta-2-microglobulin; | -0.41 | -0.84 | -0.11 | -1.68 | -0.76 | 0.68 | 0.088 |
| Beta-galactosidase; | -0.41 | -1.27 | -0.70 | -0.40 | -0.70 | 0.41 | 0.031 |
| Beta-hexosaminidase subunit alpha; | -0.59 | -1.32 |  | -1.12 | -1.01 | 0.59 | 0.084 |
| Calcium-binding mitochondrial carrier protein Aralar2; | -0.87 | -1.11 | -1.06 | -0.70 | -0.94 | 0.19 | 0.002 |
| Calcium-binding mitochondrial carrier protein SCaMC-1; | -0.99 | -0.55 | -0.54 | -0.80 | -0.72 | 0.22 | 0.005 |
| Carnitine O-palmitoyltransferase 2, mitochondrial; | -0.90 | -0.92 | -0.27 | -1.35 | -0.86 | 0.44 | 0.023 |
| Cathepsin D; | -0.28 | -0.84 | -0.99 | -1.31 | -0.86 | 0.43 | 0.022 |
| CDGSH iron-sulfur domain-containing protein 1; | -0.73 | -0.71 |  | -0.96 | -0.80 | 0.42 | 0.065 |
| Chitinase domain-containing protein 1; | -0.78 | -0.84 |  | -0.81 | -0.81 | 0.41 | 0.061 |
| Chromosome alignment-maintaining phosphoprotein 1; | -0.68 | -1.04 | -0.35 | -0.77 | -0.71 | 0.28 | 0.011 |
| Citrate synthase, mitochondrial; | -0.67 | -0.47 | -1.09 | -0.21 | -0.61 | 0.37 | 0.032 |
| Coiled-coil-helix-coiled-coil-helix domain-containing protein 6, mitochondrial; | -0.73 | -0.84 |  | -0.35 | -0.64 | 0.38 | 0.080 |
| Cytochrome b reductase 1; | -1.26 |  | -1.85 | -2.10 | -1.74 | 0.94 | 0.077 |
| Cytochrome b-c1 complex subunit 1, mitochondrial; | -0.91 | -0.65 | -0.64 | -0.65 | -0.71 | 0.13 | 0.001 |
| Cytochrome b-c1 complex subunit 6, mitochondrial; | -0.43 | -0.54 | -1.11 | -0.58 | -0.67 | 0.30 | 0.015 |
| Cytochrome b-c1 complex subunit 7; | -0.47 | -0.39 | -1.26 | -0.98 | -0.78 | 0.42 | 0.025 |
| Cytochrome b-c1 complex subunit Rieske, mitochondrial; | -0.58 |  | -0.70 | -0.51 | -0.60 | 0.31 | 0.061 |
| Cytochrome c oxidase protein 20 homolog; | -0.93 | -0.79 | -0.51 | -0.47 | -0.68 | 0.22 | 0.006 |
| Cytochrome c oxidase subunit 7A-related protein, mitochondrial; | -0.83 | -0.48 | -0.20 | -0.82 | -0.58 | 0.30 | 0.021 |
| Dehydrogenase/reductase SDR family member 7; | -0.61 | -1.67 | -0.71 | -0.14 | -0.78 | 0.64 | 0.072 |
| Delta(14)-sterol reductase; | -1.34 | -1.72 | -1.71 | -2.81 | -1.90 | 0.64 | 0.008 |
| Delta(3,5)-Delta(2,4)-dienoyl-CoA isomerase, mitochondrial; | -0.79 | -1.45 | -1.20 | -1.05 | -1.12 | 0.28 | 0.003 |
| Delta-1-pyrroline-5-carboxylate dehydrogenase, mitochondrial; | -1.26 | -0.93 | -0.88 | -0.29 | -0.84 | 0.40 | 0.019 |
| Dihydrolipoyl dehydrogenase, mitochondrial; | -1.13 | -0.81 | -0.71 | -0.60 | -0.81 | 0.23 | 0.004 |
| Dipeptidyl peptidase 2; | -0.79 | -1.25 | -0.21 | -0.88 | -0.78 | 0.43 | 0.027 |
| Disintegrin and metalloproteinase domain-containing protein 10; | -0.91 | -0.84 |  | -1.28 | -1.01 | 0.54 | 0.072 |
| Electron transfer flavoprotein subunit alpha, mitochondrial; | -0.99 | -1.02 | -0.77 | -0.38 | -0.79 | 0.30 | 0.009 |
| Electron transfer flavoprotein subunit beta; | -0.81 | -0.76 | -0.74 | -0.17 | -0.62 | 0.30 | 0.018 |
| Electron transfer flavoprotein-ubiquinone oxidoreductase, mitochondrial; | -1.29 | -1.35 |  | -1.00 | -1.21 | 0.63 | 0.069 |
| Endoplasmic reticulum metallopeptidase 1; | -1.15 | -1.46 | -0.91 | -0.09 | -0.90 | 0.59 | 0.043 |
| Endoplasmic reticulum resident protein 29; | -0.29 | -0.89 | -1.01 | -0.62 | -0.70 | 0.32 | 0.016 |
| Enoyl-CoA delta isomerase 1, mitochondrial; | -0.52 | -1.70 | -1.53 | -0.79 | -1.14 | 0.57 | 0.023 |
| Enoyl-CoA hydratase, mitochondrial; | -1.18 | -0.61 | -1.03 | -0.41 | -0.81 | 0.36 | 0.015 |
| Envoplakin; |  | -0.62 | -0.60 | -0.55 | -0.59 | 0.30 | 0.057 |
| Epoxide hydrolase 1; | -1.15 | -1.34 | -2.03 | -1.37 | -1.47 | 0.38 | 0.004 |
| ER membrane protein complex subunit 10; | -0.66 | -1.22 | -0.23 | -0.43 | -0.64 | 0.43 | 0.042 |
| ER membrane protein complex subunit 4; | -0.89 | -0.67 | -0.44 | -0.62 | -0.66 | 0.19 | 0.004 |
| ES1 protein homolog, mitochondrial; | -1.23 | -1.19 | -1.36 | -0.65 | -1.11 | 0.31 | 0.005 |
| Estradiol 17-beta-dehydrogenase 11; | -0.39 | -0.96 | -0.32 | -0.75 | -0.61 | 0.30 | 0.019 |
| Ferritin heavy chain; | -0.39 | -0.54 | -1.24 | -0.83 | -0.75 | 0.37 | 0.020 |
| Galectin-3-binding protein; | -1.50 | -1.38 | -1.70 | -1.95 | -1.63 | 0.25 | 0.001 |
| Ganglioside-induced differentiation-associated protein 1; | -1.57 |  | -1.25 | -0.52 | -1.11 | 0.71 | 0.098 |
| Glucosylceramidase; | -0.84 | -0.74 | -0.83 | -1.30 | -0.93 | 0.25 | 0.004 |
| Glutamate dehydrogenase 1, mitochondrial; | -0.81 | -0.76 | -0.68 | -0.50 | -0.69 | 0.14 | 0.001 |
| Glutathione S-transferase kappa 1; | -0.40 | -0.84 | -1.15 | -0.34 | -0.68 | 0.38 | 0.027 |
| Glyoxylate reductase/hydroxypyruvate reductase; | -0.29 | -0.53 | -0.65 | -1.03 | -0.63 | 0.31 | 0.019 |
| GTP:AMP phosphotransferase AK3, mitochondrial; | -0.96 | -1.02 | -1.36 | -0.87 | -1.05 | 0.21 | 0.002 |
| Guanine nucleotide-binding protein G(i) subunit alpha-2; | -1.46 | -0.35 | -0.20 | -1.02 | -0.76 | 0.59 | 0.063 |
| Guanine nucleotide-binding protein G(I)/G(S)/G(O) subunit gamma-12; | -0.88 | -0.15 | -0.68 | -1.22 | -0.73 | 0.45 | 0.035 |
| Guanine nucleotide-binding protein G(I)/G(S)/G(T) subunit beta-1; | -0.91 | -1.39 | -0.60 | -0.77 | -0.92 | 0.34 | 0.009 |
| Guanine nucleotide-binding protein G(q) subunit alpha; |  | -0.48 | -0.67 | -0.93 | -0.69 | 0.39 | 0.074 |
| Guanylate kinase; | -0.29 | -1.31 | -1.55 | -0.83 | -1.00 | 0.56 | 0.030 |
| H/ACA ribonucleoprotein complex subunit 4; | -0.56 | -0.42 | -0.81 | -1.32 | -0.78 | 0.40 | 0.022 |
| Haloacid dehalogenase-like hydrolase domain-containing protein 3; | -1.13 | -1.16 |  | -0.93 | -1.07 | 0.55 | 0.066 |
| Heterochromatin protein 1-binding protein 3; | -0.74 | -0.44 | -0.32 | -0.89 | -0.60 | 0.26 | 0.013 |
| Heterogeneous nuclear ribonucleoprotein H2; | -0.63 | -0.81 | -0.49 | -0.70 | -0.66 | 0.13 | 0.002 |
| Heterogeneous nuclear ribonucleoprotein U-like protein 2; | -1.10 | -1.00 | -0.81 | -0.87 | -0.95 | 0.13 | 0.001 |
| Heterogeneous nuclear ribonucleoproteins C1/C2; | -0.94 | -0.91 | -0.16 | -0.54 | -0.64 | 0.37 | 0.028 |
| HIG1 domain family member 2A, mitochondrial; | -1.28 |  | -0.50 | -1.04 | -0.94 | 0.57 | 0.089 |
| Hydroxyacyl-coenzyme A dehydrogenase, mitochondrial; | -0.16 | -0.82 | -0.67 | -0.68 | -0.58 | 0.29 | 0.019 |
| Hydroxysteroid dehydrogenase-like protein 2; | -0.73 | -1.37 | -0.49 | -0.35 | -0.74 | 0.45 | 0.035 |
| Isochorismatase domain-containing protein 2, mitochondrial; | -1.20 | -1.00 | -1.21 | -0.45 | -0.97 | 0.36 | 0.010 |
| Isocitrate dehydrogenase [NAD] subunit beta, mitochondrial; | -0.93 | -0.81 | -0.64 | -0.17 | -0.64 | 0.33 | 0.022 |
| Isoleucine--tRNA ligase, mitochondrial; | -0.91 | -0.46 | -1.01 | -0.24 | -0.66 | 0.37 | 0.026 |
| Isovaleryl-CoA dehydrogenase, mitochondrial; | -1.33 |  | -0.71 | -0.64 | -0.89 | 0.54 | 0.088 |
| Leukocyte surface antigen CD47; | -0.35 | -0.48 | -0.52 | -1.49 | -0.71 | 0.53 | 0.055 |
| Lipoamide acyltransferase component of branched-chain alpha-keto acid dehydrogenase complex, mitochondrial; | -1.39 | -1.52 |  | -0.65 | -1.19 | 0.71 | 0.088 |
| Lipolysis-stimulated lipoprotein receptor; |  | -1.68 | -1.61 | -0.79 | -1.36 | 0.79 | 0.086 |
| Long-chain fatty acid transport protein 1; | -1.59 | -0.88 | -0.83 | -0.86 | -1.04 | 0.37 | 0.009 |
| Lysophospholipase-like protein 1; | -0.53 | -0.38 | -1.24 | -0.22 | -0.59 | 0.45 | 0.055 |
| Lysophospholipid acyltransferase 5; | -0.88 | -0.95 | -0.28 |  | -0.70 | 0.46 | 0.096 |
| Lysophospholipid acyltransferase 7; | -0.76 | -0.63 | -0.53 | -0.66 | -0.65 | 0.09 | 0.001 |
| Lysosome membrane protein 2; | -1.04 | -0.42 | -0.84 | -1.11 | -0.85 | 0.31 | 0.009 |
| Lysosome-associated membrane glycoprotein 1; | -0.44 | 0.06 | -1.57 | -1.17 | -0.78 | 0.73 | 0.096 |
| Major facilitator superfamily domain-containing protein 10; | -0.34 | -1.04 | -0.62 | -1.09 | -0.77 | 0.36 | 0.017 |
| Malate dehydrogenase, mitochondrial; | -0.74 | -0.80 | -0.70 | -0.50 | -0.69 | 0.13 | 0.001 |
| Malonyl-CoA-acyl carrier protein transacylase, mitochondrial; | -0.85 |  | -0.52 | -0.55 | -0.64 | 0.35 | 0.069 |
| MARCKS-related protein; | -0.11 | -0.68 | -1.01 | -0.72 | -0.63 | 0.38 | 0.031 |
| Medium-chain specific acyl-CoA dehydrogenase, mitochondrial; | -1.20 | -0.94 | -1.21 | -1.13 | -1.12 | 0.13 | 0.000 |
| Membrane-associated progesterone receptor component 2; | -0.58 | -1.03 | -0.84 | -0.60 | -0.76 | 0.21 | 0.004 |
| Metaxin-2; | -1.10 |  | -0.94 | -0.39 | -0.81 | 0.51 | 0.091 |
| Methylcrotonoyl-CoA carboxylase beta chain, mitochondrial; | -0.88 | -1.12 | -1.19 | -0.46 | -0.91 | 0.33 | 0.009 |
| Methylcrotonoyl-CoA carboxylase subunit alpha, mitochondrial; | -1.64 | -1.92 | -0.53 | -1.51 | -1.40 | 0.60 | 0.016 |
| Methylmalonyl-CoA mutase, mitochondrial; | -0.64 |  | -0.74 | -1.08 | -0.82 | 0.45 | 0.073 |
| Microsomal glutathione S-transferase 3; | -0.37 | -1.34 | -3.87 | -2.85 | -2.11 | 1.56 | 0.066 |
| Mitochondrial fission 1 protein; | -0.28 | -0.60 | -0.75 | -1.01 | -0.66 | 0.30 | 0.016 |
| Mitochondrial import inner membrane translocase subunit Tim13; | -0.67 |  | -0.80 | -0.30 | -0.59 | 0.36 | 0.083 |
| Mitochondrial import inner membrane translocase subunit Tim9; | -0.67 | -0.67 | -0.91 | -0.99 | -0.81 | 0.16 | 0.002 |
| Mycophenolic acid acyl-glucuronide esterase, mitochondrial; | -0.72 | -0.54 | -0.24 | -0.93 | -0.61 | 0.29 | 0.017 |
| NAD-dependent malic enzyme, mitochondrial; | -0.63 | -0.54 | -0.96 | -0.40 | -0.63 | 0.24 | 0.009 |
| NADH dehydrogenase [ubiquinone] 1 alpha subcomplex assembly factor 4; | -0.56 | -0.58 |  | -0.65 | -0.60 | 0.30 | 0.058 |
| NADH dehydrogenase [ubiquinone] 1 alpha subcomplex subunit 12; | -1.13 | -1.04 | -0.84 | -0.34 | -0.84 | 0.35 | 0.013 |
| NADH dehydrogenase [ubiquinone] 1 alpha subcomplex subunit 2; | -0.62 | -0.81 | -0.85 | -1.06 | -0.84 | 0.18 | 0.002 |
| NADH dehydrogenase [ubiquinone] 1 alpha subcomplex subunit 5; | -0.67 |  | -1.06 | -0.68 | -0.80 | 0.44 | 0.072 |
| NADH dehydrogenase [ubiquinone] 1 alpha subcomplex subunit 9, mitochondrial; | -0.65 | -0.46 | -0.83 | -0.70 | -0.66 | 0.15 | 0.002 |
| NADH dehydrogenase [ubiquinone] 1 beta subcomplex subunit 10; | -0.75 | -0.42 | -0.83 | -0.85 | -0.71 | 0.20 | 0.004 |
| NADH dehydrogenase [ubiquinone] 1 beta subcomplex subunit 11, mitochondrial; | -0.61 | -0.37 | -1.17 | -1.03 | -0.80 | 0.37 | 0.017 |
| NADH dehydrogenase [ubiquinone] 1 beta subcomplex subunit 4; | -0.69 | -0.40 | -0.78 | -0.71 | -0.65 | 0.17 | 0.003 |
| NADH dehydrogenase [ubiquinone] 1 beta subcomplex subunit 7; | -0.59 | -0.23 | -0.97 | -0.78 | -0.64 | 0.32 | 0.019 |
| NADH dehydrogenase [ubiquinone] 1 beta subcomplex subunit 8, mitochondrial; | -0.52 | -1.38 | -1.79 | -1.44 | -1.28 | 0.54 | 0.015 |
| NADH dehydrogenase [ubiquinone] 1 beta subcomplex subunit 9; | -0.38 | -0.80 | -0.81 | -1.36 | -0.84 | 0.40 | 0.019 |
| NADH dehydrogenase [ubiquinone] iron-sulfur protein 2, mitochondrial; | -0.79 | -0.84 | -0.45 | -0.49 | -0.64 | 0.20 | 0.005 |
| NADH dehydrogenase [ubiquinone] iron-sulfur protein 5; | -1.08 | -0.46 | -0.64 | -0.43 | -0.65 | 0.30 | 0.016 |
| NADH dehydrogenase [ubiquinone] iron-sulfur protein 6, mitochondrial; | -0.51 | -1.07 | -0.88 | -0.18 | -0.66 | 0.40 | 0.032 |
| NADH-cytochrome b5 reductase 1; | -0.69 | -1.04 | -1.13 | -0.78 | -0.91 | 0.21 | 0.002 |
| Neuroplastin; | -0.22 | -0.87 | -0.39 | -1.42 | -0.73 | 0.54 | 0.056 |
| NHP2-like protein 1; | -0.22 | -0.98 | -0.96 | -1.54 | -0.93 | 0.54 | 0.033 |
| Nicastrin; | -0.43 | -0.18 | -0.81 | -1.12 | -0.64 | 0.41 | 0.039 |
| Non-specific lipid-transfer protein; | -0.81 | -1.74 | -0.91 | 0.03 | -0.86 | 0.72 | 0.078 |
| Nuclear mitotic apparatus protein 1; | -0.54 | -1.19 | -0.09 | -1.24 | -0.77 | 0.55 | 0.053 |
| Nucleolar protein 56; | -0.61 | -0.67 |  | -0.89 | -0.72 | 0.38 | 0.066 |
| Nucleolar protein 58; | -0.33 | -0.61 | -0.80 | -1.22 | -0.74 | 0.37 | 0.021 |
| O-acetyl-ADP-ribose deacetylase MACROD1; | -0.65 | -0.96 |  | -0.29 | -0.63 | 0.42 | 0.095 |
| Oligosaccharyltransferase complex subunit OSTC; | -0.41 | -1.25 | -1.14 | -1.39 | -1.05 | 0.44 | 0.014 |
| Peroxisomal acyl-coenzyme A oxidase 3; | -0.62 |  | -1.20 | -1.31 | -1.04 | 0.60 | 0.082 |
| Peroxisomal membrane protein 11B; | -0.30 | -0.49 | -0.81 | -0.85 | -0.61 | 0.26 | 0.013 |
| Peroxisomal membrane protein PEX14; | -0.77 | -1.48 | -0.29 | -1.10 | -0.91 | 0.50 | 0.028 |
| Persulfide dioxygenase ETHE1, mitochondrial; |  | -0.67 | -0.48 | -0.64 | -0.60 | 0.31 | 0.061 |
| Phospholipase D3; | -0.41 | -0.86 |  | -0.62 | -0.63 | 0.36 | 0.075 |
| Piezo-type mechanosensitive ion channel component 1; | -0.44 | -0.92 |  | -1.23 | -0.86 | 0.54 | 0.092 |
| PRA1 family protein 3; | -1.05 | -0.84 | -0.37 | -0.82 | -0.77 | 0.29 | 0.009 |
| Probable cation-transporting ATPase 13A1; | -0.66 | -0.85 | -0.39 | -0.99 | -0.72 | 0.26 | 0.008 |
| Probable lysosomal cobalamin transporter; | -0.30 | -1.27 | -1.40 | -1.64 | -1.15 | 0.59 | 0.024 |
| Prohibitin; | -0.51 | -0.54 | -0.71 | -0.62 | -0.60 | 0.09 | 0.001 |
| Prohibitin-2; | -0.52 | -0.46 | -0.80 | -0.55 | -0.58 | 0.15 | 0.003 |
| Propionyl-CoA carboxylase alpha chain, mitochondrial; | -1.17 | -1.27 | -0.77 | -0.76 | -0.99 | 0.27 | 0.004 |
| Propionyl-CoA carboxylase beta chain, mitochondrial; | -0.97 | -1.93 | -0.80 | -1.07 | -1.19 | 0.50 | 0.015 |
| Protein canopy homolog 3; | -0.55 | -1.22 | -0.24 | -1.30 | -0.83 | 0.52 | 0.038 |
| Protein FAM134A; | -0.81 | -1.32 |  | -0.82 | -0.98 | 0.55 | 0.076 |
| Protein NipSnap homolog 1; | -1.23 | -0.55 | -1.06 | -0.68 | -0.88 | 0.32 | 0.009 |
| Protein NipSnap homolog 2; | -1.06 | -0.92 | -0.91 | -0.58 | -0.87 | 0.20 | 0.003 |
| Protein NipSnap homolog 3A; | -0.50 | -0.95 | -1.32 |  | -0.92 | 0.57 | 0.091 |
| Protein S100-P; |  | -2.16 | -1.20 | -1.35 | -1.57 | 0.89 | 0.084 |
| Protoporphyrinogen oxidase; | -0.67 | -0.76 |  | -1.26 | -0.90 | 0.52 | 0.080 |
| Putative oxidoreductase GLYR1; | -0.85 | -0.39 | -0.83 | -0.40 | -0.62 | 0.26 | 0.012 |
| Pyruvate dehydrogenase E1 component subunit alpha, somatic form, mitochondrial; | -0.34 | -0.83 | -1.02 | -0.40 | -0.65 | 0.33 | 0.021 |
| Pyruvate dehydrogenase E1 component subunit beta, mitochondrial; | -0.96 | -1.06 | -0.69 | 0.12 | -0.65 | 0.54 | 0.070 |
| Quinone oxidoreductase; | -0.44 | -1.09 | -0.76 | -0.33 | -0.66 | 0.34 | 0.022 |
| Rab-like protein 3; | -0.51 | -0.41 |  | -1.02 | -0.65 | 0.42 | 0.092 |
| Ragulator complex protein LAMTOR1; | -0.72 | -0.58 | -1.50 | -1.11 | -0.98 | 0.41 | 0.014 |
| Ragulator complex protein LAMTOR3; | -0.69 | -0.81 | -1.52 | -1.37 | -1.10 | 0.41 | 0.010 |
| Ragulator complex protein LAMTOR5; | -0.64 | -0.54 | -1.69 | -0.77 | -0.91 | 0.53 | 0.032 |
| Ras-related GTP-binding protein C; | -0.91 | -0.41 | -1.16 | -0.88 | -0.84 | 0.31 | 0.009 |
| Ras-related protein Rap-1A; | -0.45 | -1.50 | -1.37 | -1.27 | -1.15 | 0.47 | 0.014 |
| Ras-related protein Rap-1b; | -0.23 | -0.51 | -1.27 | -0.57 | -0.65 | 0.44 | 0.044 |
| Receptor-type tyrosine-protein phosphatase kappa; | -1.23 | -1.64 | -0.77 | -0.39 | -1.01 | 0.54 | 0.027 |
| rRNA 2'-O-methyltransferase fibrillarin; | -0.64 | -0.56 | -0.71 | -0.89 | -0.70 | 0.14 | 0.001 |
| Saccharopine dehydrogenase-like oxidoreductase; | -0.48 | -0.95 | -0.66 | -0.87 | -0.74 | 0.21 | 0.004 |
| Sepiapterin reductase; | -0.26 | -0.61 | -1.26 | -0.82 | -0.74 | 0.42 | 0.028 |
| Serine--tRNA ligase, mitochondrial; | -0.85 | -0.58 | -0.59 | -0.83 | -0.71 | 0.15 | 0.002 |
| Succinyl-CoA ligase [ADP/GDP-forming] subunit alpha, mitochondrial; | -0.81 | -0.91 | -0.71 | -0.22 | -0.66 | 0.31 | 0.016 |
| Succinyl-CoA ligase [ADP-forming] subunit beta, mitochondrial; | -0.97 | -0.22 | -1.06 | -0.18 | -0.61 | 0.47 | 0.059 |
| Tapasin; | -0.27 | -0.70 |  | -0.91 | -0.63 | 0.41 | 0.094 |
| Target of Myb protein 1; |  | -1.53 | -0.87 | -0.71 | -1.04 | 0.63 | 0.089 |
| Thioredoxin-dependent peroxide reductase, mitochondrial; | -0.73 | -1.32 | -1.63 | -0.78 | -1.12 | 0.44 | 0.012 |
| Thiosulfate sulfurtransferase; | -0.84 | -1.12 | -0.53 | -1.25 | -0.94 | 0.32 | 0.008 |
| Transcription elongation factor SPT6; | -0.84 | -1.02 |  | -0.35 | -0.74 | 0.46 | 0.090 |
| Transmembrane protein 11, mitochondrial; | -0.79 |  | -1.36 | -1.47 | -1.21 | 0.67 | 0.079 |
| Transmembrane protein 120A; | -0.89 | -0.57 |  | -0.71 | -0.72 | 0.38 | 0.067 |
| Transmembrane protein 126A; | -0.79 | -1.02 | -1.87 | -0.53 | -1.05 | 0.58 | 0.029 |
| Transmembrane protein 192; | -0.57 |  | -1.65 | -1.14 | -1.12 | 0.71 | 0.098 |
| Transmembrane protein 205; | -0.41 | -0.49 | -1.19 | -0.97 | -0.77 | 0.38 | 0.020 |
| Transmembrane protein 245; | -0.70 |  | -0.78 | -1.17 | -0.88 | 0.49 | 0.074 |
| Transmembrane protein 256; | -0.29 | -0.91 |  | -0.82 | -0.67 | 0.43 | 0.092 |
| Transmembrane protein 87B; | -0.45 | -0.70 |  | -1.18 | -0.78 | 0.49 | 0.092 |
| Tricarboxylate transport protein, mitochondrial; | -0.99 | -0.97 | -0.94 | -0.73 | -0.91 | 0.12 | 0.000 |
| Trifunctional enzyme subunit alpha, mitochondrial; | -0.94 | -0.58 | -1.21 | -0.87 | -0.90 | 0.26 | 0.005 |
| Trifunctional enzyme subunit beta, mitochondrial; | -0.99 | -1.11 | -1.34 | -1.12 | -1.14 | 0.15 | 0.000 |
| Trimethyllysine dioxygenase, mitochondrial; | -1.17 |  | -1.58 | -1.29 | -1.35 | 0.69 | 0.070 |
| Tripeptidyl-peptidase 1; | -0.84 | -1.83 | -0.68 | -1.26 | -1.15 | 0.51 | 0.017 |
| Trypsin-1; | -1.61 | -1.89 | -2.31 | -3.11 | -2.23 | 0.65 | 0.006 |
| Type 1 phosphatidylinositol 4,5-bisphosphate 4-phosphatase; | -0.97 | -0.97 | -2.10 | -1.37 | -1.35 | 0.53 | 0.012 |
| Ubiquinol-cytochrome c reductase complex chaperone CBP3 homolog; | -0.75 | -0.92 | -1.09 | -0.85 | -0.90 | 0.14 | 0.001 |
| Ubiquinone biosynthesis protein COQ9, mitochondrial; | -0.85 | -0.62 |  | -0.96 | -0.81 | 0.43 | 0.068 |
| Up-regulated during skeletal muscle growth protein 5; | -0.74 | -0.94 | -1.75 | -0.88 | -1.08 | 0.46 | 0.014 |
| Valacyclovir hydrolase; | -1.02 | -0.86 |  | -1.44 | -1.11 | 0.60 | 0.075 |
| Vesicle-associated membrane protein 7; | -1.07 | -0.65 |  | -0.37 | -0.70 | 0.45 | 0.094 |
| VIP36-like protein; | -0.70 | -0.40 |  | -0.81 | -0.64 | 0.36 | 0.073 |

Shown is a comprehensive listing of all proteins which are differentially down-regulated in minimum of three AMPAC cell lines (AMP7, AVC1, RCB1280 and SNU487) with fold change of at least 50% (log_2_ ratio of < -0.58, *p*-value < 0.1, two-tailed t-test).
